# Advantages and disadvantages of mobile applications for workplace health promotion: A scoping review

**DOI:** 10.1371/journal.pone.0296212

**Published:** 2024-01-02

**Authors:** Maren Junker, Markus Böhm, Helmut Krcmar

**Affiliations:** 1 Department of Informatics, Technical University of Munich, Munich, Bavaria, Germany; 2 Department of Informatics, University of Applied Sciences Landshut, Landshut, Germany; Teesside University, UNITED KINGDOM

## Abstract

Different interventions and methods are used for workplace health promotion (WHP) programmes, including mobile applications (apps), which have proven effective among different health outcomes if properly communicated and developed. However, knowledge is lacking on the potential advantages and disadvantages of using this technology for WHP compared with nontechnical WHP programmes to support employers in their decision making and effective development of such an intervention. To obtain an overview of factors that decision-makers should consider when deciding whether to implement an WHP app, we conducted a scoping review of studies that have evaluated WHP apps. Potential advantages and disadvantages of using mobile apps for WHP were summarised using a strengths, weaknesses, opportunities and threats (SWOT) analysis. Articles were included if they focussed on a WHP app, were published between 2007 and 2022 in German or English, and evaluated an app for the general employee population. Altogether, 38 studies were included in the review, demonstrating WHP apps’ effectiveness among various use cases in terms of content, e.g., mindfulness or sleep, and target groups, e.g., office workers, nurses or pilots. Strengths were found in the context of adoption, convenience for users, the targeted employee group’s reach and cost-effectiveness. However, the review also identified some disadvantages in apps, including technical difficulties and usage barriers, as well as challenges, e.g., privacy issues and maintenance costs. Generally, our review found that different factors need to be considered when deciding whether to implement a WHP app based on the individual company situation, e.g., shift work, content to be communicated, and expectations for health parameter screening, among many others. By summarising recent literature on WHP apps, this review uses scientific knowledge to give employers an overview of potential factors to consider in their decision making.

## Background

Because a high proportion of the general adult population works, companies are viewed as effective settings for health promotion; thus, they increasingly are the focus of health promotion and health behaviour change programmes [1]. According to the World Health Organisation (WHO), workplace health promotion (WHP) is part of occupational health management, which ‘deals with all aspects of health and safety in the workplace and has a strong focus on primary prevention of hazards’ [2].

Effective WHP offers advantages for a variety of actors. First, the government benefits because targeting the workplace means that a high proportion of the adult population with varying demographic backgrounds and health statuses can be reached [1]. Second, employees save money, time and effort, e.g., by having access to workout facilities or medical checkups at their place of work or practising particular exercises tailored to the nature of their work. Finally, employers benefit because absenteeism rates are reduced, and employees are more productive [3, 4].

Despite WHP’s theoretical potential, a literature review postulated that only evidence-based programmes can meet target achievements, e.g., improved health or financial advantages, which is often not the case [5]. Furthermore, a literature review of general WHP programmes’ participation rates supports the need for more research into their acceptance and factors that determine participation, as participation rates are often not reported or are below 50% [6]. Robroek et al. (2021) found that general WHP programmes mostly do not meet expected outcomes; thus, new approaches are necessary. Aside from strengthening the need for a holistic perspective on the ‘system’, including working cultures–which fall outside of this study’s scope–the authors emphasise that a more targeted approach with personalised interventions and a new perspective on implementation strategies are necessary to reach the right target group and develop an effective WHP programme [7]. In particular, reaching target groups in lower socioeconomic positions as well as those working in difficult workplace conditions, e.g., shift work, needs to be considered. Thus, employers need to make informed decisions about their WHP programmes’ approach, content and format to make them truly effective [8].

One solution to this may be to use mobile technologies, which can be tailored to specific needs and combine various interventions in one app [9]. WHP representatives have started to rely on technology, particularly mobile applications (apps) [10]. The Global Observatory for eHealth (GOe) defines this so-called *mobile health* (mHealth) as ‘medical and public health practice supported by mobile devices, e.g., mobile phones, patient-monitoring devices, personal digital assistants and other wireless devices’ [11]. mHealth has proven to be effective for various health outcomes and has been used for self-assessment and improvement in physical activity in a variety of health fields.

Various literature reviews indicate that many studies have attempted to evaluate whether apps are effective in general. For example, Buckingham et al. (2019) examined the literature on mHealth use (particularly health apps) for improving physical activity and reducing sedentary time in the workplace. The researchers found that such apps generally were effective and accepted, but that usage dropped quickly over time [12]. Furthermore, a 2021 review by Chandrasekaran et al., which mapped free WHP apps for physical activity and sedentary behaviour to behavioural change techniques (BCTs), found that only a few BCTs were applied and that most apps were not using BCTs to develop effective WHP apps. In particular, social support and gamification elements were lacking in apps [13]. Another study also found infrequent use of BCTs and further demonstrated that most apps have elicited safety and security concerns [13].

Not much general research on WHP apps has been conducted, as most extant studies have tested individual mHealth apps’ effectiveness and derived advantages as a means of WHP, with little guidance given to employers in terms of factors they should consider when considering implementation of mHealth programmes (5,20).

One advantage of these apps is that they can be adapted to target groups and individualised to suit specific customers; thus, they can reach diverse groups [14, 15]. Other advantages include self-monitoring, management, provision of information independent of time and location, and the option of adjusting interventions to suit personal and organisational needs. It has been argued that these advantages are particularly beneficial today, when new ways of working are becoming prevalent [16, 17] and support the aims of Robroek et al. (2021), who also tailored interventions to reach target groups outside of office workers. With these advantages, mHealth also could be applicable to new ways of working (e.g., by reaching working populations in different locations) [18].

When looking into these studies, it seems that apps can be effective and advantageous compared with nontechnical solutions in some contexts and under certain conditions, particularly when using evidence-based approaches and BCTs. However, before deciding what an app should look like, decision-makers need to vote for or against such technology in general. To date, these apps’ advantages and disadvantages generally have not been summarised so that employers can map this information to their companies’ individual situations and make informed decisions. Developers could use the factors that developers used to ensure they concentrate on the right factors when developing such apps.

While combining research and practice was mentioned as one recommendation in a recent study [13], the present study aims to provide an overview of factors that might influence companies’ decision-makers. This study’s aim is unique because it does not concentrate on individual interventions’ effectiveness in certain health outcomes, nor does it provide a theoretical framework for potential acceptance of an WHP app. Instead, we analyse the strengths, weaknesses, opportunities and threats of using technology for WHP compared with other interventions. Therefore, the study also addresses other points that might be advantageous when using such technology, e.g., enhancing employees’ curiosity, which can strengthen productivity. This study addresses the following research questions:

1. What are the reasons for using (strengths and opportunities) WHP apps for the general working population compared with nontechnical supported interventions?
2. What are the reasons against using (weaknesses and threats) WHP apps for the general working population compared with nontechnical supported interventions?

A strengths, weaknesses, opportunities, and threats (SWOT) analysis was used to organise the results and evaluate workplace mobile health interventions’ potential compared with nontechnical interventions from the perspective of companies’ decision-makers.

## Methods

To answer the research questions, a scoping review was conducted. This theoretical approach was chosen to cover different perspectives, workplace settings and study methods to derive relevant factors. This methodology was chosen because some companies have started using mHealth for WHP and have reported their results in scientific studies, including arguments for and against mHealth, as well as experiences with this technology. To provide employers with guidelines to decide whether apps could be a solution for their companies, we wanted to summarise WHP apps’ advantages and disadvantages compared with nontechnical interventions for WHP using the SWOT analysis method.

Considering that this study includes a rather broad search of studies, a scoping review, rather than a concrete literature review, was conducted based on the guidelines described in the PRISMA Extension for Scoping Reviews (PRISMA-ScR), which was developed by experts and published in 2018 [19, 20]. This structured approach was followed except for individual exceptions as explained in the limitations section. The PubMed, PsycInfo and Google Scholar databases were used. This rather broad approach was chosen because the topic combines different fields of research, e.g., technology, economics and health sciences. The search covered articles that examined health apps in the workplace published between 2007 and the end of 2022 that adhere to the exclusion and inclusion criteria. The last search was conducted on January 6, 2023, to ensure that all articles from 2022 were included.

To answer the research questions and examine only studies addressing mHealth, the following inclusion criteria were used:

1. Studies focussing on WHP
2. Use of a smartphone app as the main channel of intervention in the study
3. Studies published later than 2007 (because apps have only been available since then)
4. Studies written in German or English

To ensure that the studies (and, thus, advantages and disadvantages) covered in our review are generalizable to different contexts, only studies focussing on apps tailored to the general working population without diseases and health issues were included. Furthermore, as many studies have focussed on supporting nurses in their jobs or safety, these studies also were excluded to strengthen the research on apps and the research gap. Thus, the following exclusion criteria were used:

1. The WHP app’s effects on a particular health outcome were not extractable because additional technologies or communication channels were used, e.g., smartphone-optimised websites or wearables as main components of the intervention
2. The use of mHealth only as a measurement tool, not as an intervention (e.g., an app used to measure sedentary time, but with no intervention included)
3. A target group other than general employees, e.g., apps for health professionals to support their daily work
4. A target group comprising employees with a specific disease (e.g., diabetes) or condition (e.g., pregnancy or highly stressed or overweight employees)
5. An emphasis on safety, rather than general health prevention and promotion
6. Literature reviews, theoretical frameworks, project reports, study protocols, study designs, app design or validation studies, theses and nonscientific publications

Studies that only described the study design and/or a launch test for an app also were excluded because the app’s effects were not extractable and, thus, derived advantages and disadvantages could not be validated. Furthermore, if the study contribution was purely theoretical and no specific app was used, then the study was excluded for the same reason (e.g., 24, 25).

First, the databases were searched using a defined set of keywords and themes. The search terms are listed in Table 1 and were combined using ‘AND’. The search terms for PubMed were: ‘(Mobile OR mHealth OR Application OR App OR App-based OR Smartphone OR Smartphone-based OR Wearable) AND (Workplace OR Workplaces OR Worksite OR worksites OR Corporate OR Manager OR Managers OR Leaders OR Leader OR Work OR Worker OR workers OR Employee OR Employees OR Working) AND (Health OR Wellness OR Stress OR Sedentary time OR Mindfulness OR Well-being OR Stress at work OR Fitness OR Work-related stress)’. For Google Scholar, the searches were done individually.

**Table 1:** Search terms used in various databases for the literature review (included years: 2007–2022).

Search terms for app	Search terms for workplace	Search terms for health
Mobile	Workplace +s	Health
mHealth	Worksite +s	Wellness
Application	Corporate	Stress
App	Manager +s	Sedentary time
App-based	Leader +s	Mindfulness
Smartphone	Work	Well-being
Smartphone-based	Worker +s	Stress at work
Technology-based	Employee +s	Fitness
Digital	Working	Work-related stress
Wearable		

All articles were transferred into a reference programme to remove duplicates. During the article transfer, it became clear that articles focussing on specific diseases, target groups other than employees or technologies other than apps still were included. Because such information often was included in the title, the authors decided to review all the titles as a first step.

Next, the researchers rated the articles’ abstracts and full texts. During the final evaluation step, the full texts were read, and the included articles’ reference lists also were evaluated (using the snowball method) to ensure that all relevant articles were included in the study [21]. For these snowball papers, the same process of scanning the abstract and rating the abstract and full text was followed.

Studies were evaluated based on the methodology used, the publishing journal or conference, interventions used, demonstrated effectiveness and the advantages and disadvantages they mentioned. After consolidating relevant information from the included studies in one Excel spreadsheet, we sorted the tables based on their study characteristics (e.g., methods used and outcome measures of effectiveness). An additional Excel spreadsheet was created to summarise the relevant SWOT factors of the apps and calculate the frequencies. Arguments for using WHP apps were categorised into strengths and opportunities, while arguments against using WHP were categorised into weaknesses and threats. Opportunities and threats included potential pitfalls or opportunities for further developments in mHealth for WHP mentioned in the paper. These mainly were extracted from the outlook sections. The results from the individual categories are described in the results section. Details about the studies also can be found in the S1 Appendix, where they were structured based on methods, journal, intervention used, effectiveness and advantages and disadvantages.

For the summary of SWOT factors, the focus was on factors that are advantageous or disadvantageous compared with other nontechnical programmes,. To organise these factors, subheadings were created: acceptance and adherence factors; functionalities and effectiveness. For the advantages also a category “employer perspective” was created.

If any studies mentioned factors found in another paper, these factors still were viewed as presenting a full picture of advantages and disadvantages, and were marked accordingly in the results tables.

## Results

**Fig 1:**
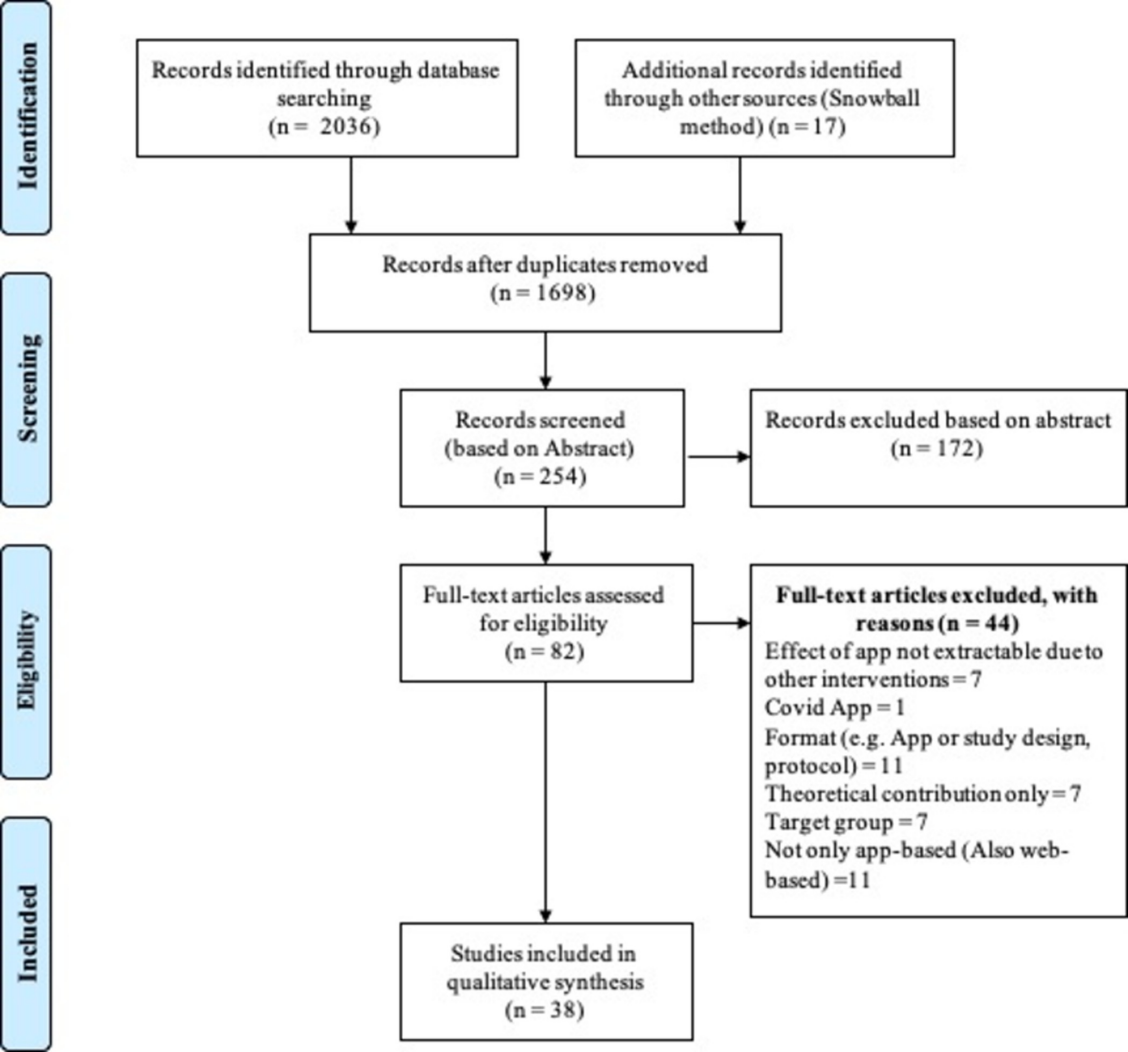
Flow diagram of the scoping review conducted for articles between 2007 and 2022.

This section briefly describes the scoping review’s results. Altogether, 1,698 titles were screened. During the second step, 254 articles’ abstracts were rated based on the inclusion and exclusion criteria, and 82 articles were accepted for full-text evaluation (Fig 1). Altogether, 38 papers ultimately were accepted for this scoping review.

### Overview of the included studies

The results from the studies varied widely due to the varying methodologies used, work contexts and target groups examined. This supported our research approach of focussing on factors that were relevant in decisions for or against using mHealth for WHP because it included different settings and perspectives. Thus, this study sheds light on individual factors that might contribute to these apps’ success or failure.

In examining the timeline, it became obvious that research had increased rapidly over time. Whereas we did not include any paper published earlier than 2013, for the years 2013, 2014 and 2016, we included only two studies per year, and from 2018 onwards, we included at least five studies per year. We included 10 studies published in 2022. In this context, technical developments over time also were observed, as the papers published earlier included less-sophisticated interventions.

**Fig 2:**
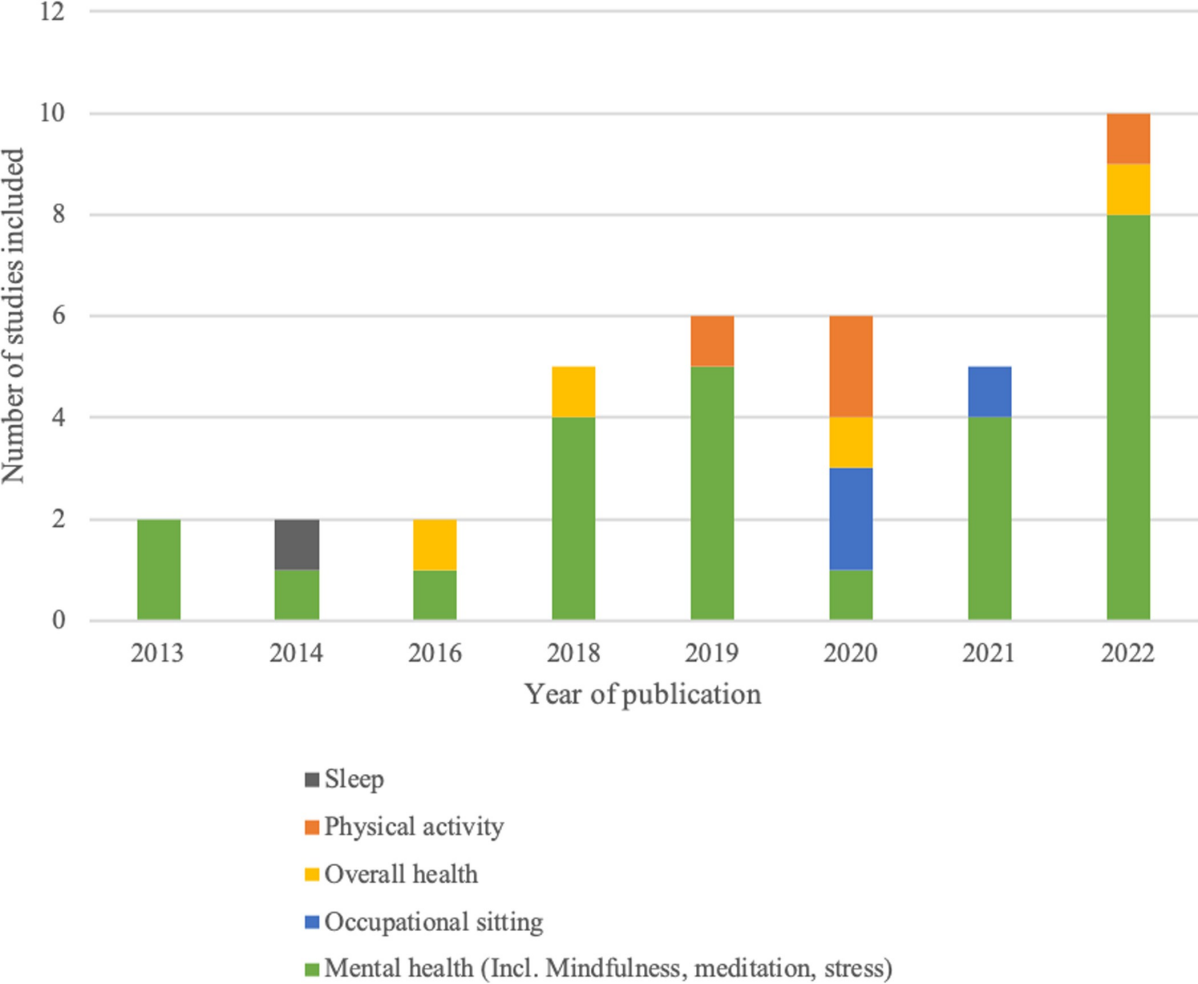
Included studies per year and theme.

Generally, the studies could be categorised based on the topic that the apps investigated: overall health; physical health apps and mental health/mindfulness apps (Fig 2 presents the categories per year of publication). Furthermore, different workplace groups were targeted (e.g., office workers, nurses, school staff and drivers). Due to the wide range of measured outcomes, the authors did not evaluate mHealth’s overall effectiveness, in line with Lee et al.’s argument that literature reviews should focus on specific areas, rather than broad topics [22]. The research outcomes are described briefly below.

The countries in which the studies were conducted differed widely: 19 studies were conducted in Europe (six in the UK); four in Asia; four in Australia; one in South America; and five in the United States. Five further studies were conducted in several countries at once.

Table 2 presents the methods used, with a clear emphasis on randomised controlled trials (RCTs). The number of participants in each study ranged from 15 to 2,946.

**Table 2:** Methods used in the included studies.

Methods used	Number of studies	References
Randomised controlled trial or quasi-RCT	22	[17, 23–43]
Case, field or intervention study	8	[44–51]
Qualitative study	3	[52–54]
Mixed-methods design	3	[9, 55, 56]
Survey study	1	[57]
Process evaluation	1	[58]

#### Measured outcomes

The measured outcomes (e.g., stress, mindfulness, objective medical screening values or physical activity) also differed, making it impossible to make judgements on the apps’ effectiveness in general. This also was due to differences in time horizons and challenges in extracting app effects, e.g., communication about a programme already might trigger health awareness [25, 58].

Most studies (27) reported positive effects in terms of targeted health outcomes, while two indicated limited effectiveness, as only short-term effects could be shown or effects could only be shown for long-term sitting [41, 48]. Two other studies indicated no additional effects among the app users [25, 58] and, therefore, concluded that the observed effects were not the result of the intervention, but of increased health awareness. For seven studies, effectiveness was not reported, as the measured outcomes for usage of the app were based on either mixed-methods or qualitative evaluations.

Studies using apps aimed at physical health behaviour had measured the effectiveness of the apps in addressing different behaviours (e.g., sedentary behaviour or steps taken per day) [48, 55, 56]. Furthermore, three studies focussed on occupational sitting only [23, 24, 49], and four adopted a comprehensive approach to health comprising various facets (physical and mental health) [9, 26, 44, 52]. One study focussed on sleep for airplane pilots [36].

Most of the apps (based on 26 studies) addressed mental health, mindfulness or stress and indicated positive outcomes in terms of stress or depressive or psychological symptoms, as well as self-rated health and workability or engagement. This is in line with WHO, which has stated that mental health support is needed in workplaces [59]. Five studies focussed on acceptance and commitment therapy, and tested therapy’s effectiveness in addressing perceived health, psychological flexibility, work engagement, mental health, well-being and stress [17, 38, 41, 46]. Only one study did not indicate positive effects [58].

Furthermore, some studies used commercially available apps (and, thus, ones not tailored to companies) to test their effectiveness among their target group. Examples of these apps are the Calm app [43, 57] and Headspace. Effectiveness in terms of, e.g., stress perception, perceived mindfulness and perceived well-being could be demonstrated [27, 34, 39, 40]. Deady et al. (2020) found that the HeadGear app decreased depressive symptoms, anxiety symptoms and sick days, though it increased self-rated work productivity and demonstrated high acceptability and utility. Positive effects were found in the areas of mindfulness, work engagement, job satisfaction, emotional exhaustion, emotional intelligence, self-efficacy [50], well-being, coping with stress and job strain, and perceptions of workplace social support [27]. Furthermore, one study compared face-to-face mindfulness-based resilience training (MBRT) with a resilience-based smartphone programme over six weeks. Both the in-person MBRT and smartphone groups demonstrated significantly increased perceived well-being [35]. Details regarding the apps’ effectiveness are provided in the S1 Appendix.

Several studies have targeted special occupational groups, e.g., van Drongelen et al. (2014) investigated fatigue among airplane pilots. In a randomised controlled trial, hypotheses on the positive effects of app use on fatigue, sleep, nutrition and physical activity were supported [36, 60]. Truckers represent another occupational group that has been addressed [52]. Due to their travel schedules, it was found that it is more difficult for truckers to implement health promotion measures than other workers, but apps could be a solution. Nine studies focussed on nurses or healthcare employees [29, 31, 33, 35, 37, 39, 41, 49, 56]. One such study, by Hwang et al. (2019), found significant changes in the experimental group in terms of stress, emotional labour, well-being and self-efficacy. Other studies focussed on public school staff [32], male-dominated industries [54], pharma employees [27], university staff [40, 46], governmental organisational staff [25, 42], remote workers [57], or entrepreneurs [53], or else included different employers [26, 38, 58]. The remaining 15 studies investigated office workers.

### Strengths, weaknesses, opportunities and threats

Our study’s purpose was to evaluate the arguments for and against using WHP apps compared with nontechnical WHP interventions, which might influence decision-makers in their consideration of WHP apps. These arguments were categorised based on a SWOT analysis. The strengths and weaknesses were structured based on acceptance, effectiveness, functionalities and employer perspective. As is typical in a scoping review, the results were summarised broadly to provide an initial overview, rather than going into detail.

During our review process, we found that the authors often mentioned some factors; however, these were not directly retrieved from their own results, but instead were references to other studies. As these factors still seemed important to these authors, we included the factors in our calculations as well. An aggregated summary of the factors is presented in Table 3 (including only factors that we mentioned more than once). The full list can be found in the S2 and S3 Appendices.

**Table 3:** Strengths, weaknesses, opportunities and threats of WHP apps *.

	Total number	N studies found that factor	N studies mentioned that factors (referenced to another study)		Total number	N studies found that factor	N studies mentioned that factors (referenced to another study)
**Strengths**				**Weaknesses**			
**Acceptance/Adherence/Use**				**Acceptance/Adherence/Use**			
Accessible	16	8	8	Lack of time for use (at work)	12	12	0
Independent of time and place	13	7	6	Short use/high attrition	7	6	1
Embedded in daily and work routines	9	3	6	Privacy and data concern	5	4	1
Convenient	5	3	2	Engagement drops over time	4	2	2
Users are curious (leads to use)	4	4	0	Additional stressors at work	3	3	0
Self-guided (learning)	4	3	1	Preferences of private apps	2	2	0
User’s autonomy	4	3	1	Social pressure	2	2	0
Ease of use	4	3	1	Lack of perceived need	2	2	0
Easy to access	3	3	0	Lack of motivation/laziness	2	2	0
Short and easy exercises	3	3	0	Gender differences	2	2	0
Less time-consuming compared with ordinary programmes	3	3	0				
Anonymity	3	2	1				
Reduces stigma	3	1	2				
Fun	2	2	0				
Short use possible/brevity	2	1	1				
Interaction with participants in daily life	2	1	1				
Simplicity	2	1	1				
User-friendly	2	1	1				
Popular technology	2	0	2				
**Functionalities**				**Functionalities**			
Reminders and notifications	10	9	1	Technical difficulties (e.g., battery or wireless connection)	6	6	0
Sharing of personal progress	2	1	1	Reminders perceived as annoying or frustrating	4	4	0
				No social interaction (e.g., for discussions)	2	2	0
				No suitable phone (all platform needs to be included)	2	2	0
**Effectiveness**				**Effectiveness**			
Personalization/customization	15	10	5	Might not be right for severe cases of mental problems (Lack of personal contact)	3	2	1
Self-monitoring (also in stressful situations)	12	8	4	Issue of measurement accuracy	3	3	0
Automatic and reliable self-monitoring (e.g., sensor-based pedometer)	6	6	0				
Real-time feedback/ecological momentary intervention	4	3	1				
Progress tracking in real time	4	1	3				
Visual feedback	2	2	0				
Insights into physical activity patterns/awareness	2	2	0				
Individual, tailored support for goals and motivation	2	1	1				
Immediate support	2	1	1				
**Employer perspective**							
Cost-effective	11	7	4				
Wide reach	10	4	6				
Wide distribution and use of smartphones in population	9	3	6				
Low costs	8	4	4				
Flexibility	5	4	1				
Adaptability	4	2	2				
Possible for workers who cannot participate in face-to-face activities, e.g., shift workers	3	2	1				
Accurate/objective measure of programme engagement in real time	3	1	2				
Integration of other WHP programmes	2	2	0				
Limits infection risk	2	2	0				
Target at workplace needs/context-aware	2	1	1				
Preventive use	2	0	2				
**Opportunities**				**Threats**			
Need to be embedded in other employee support package parts	2	2	0	Lack of experimental/ scientific evidence	8	5	3
Connection to other technical devices possible, e.g., wearables	2	2	0	Limited sustainability of a smartphone intervention	2	2	0
Additional functionalities such as gamification, challenges, messages or organizational engagement	2	2	0				

*(Only factors found more than once are included. For the full list, please see S2 and S3 Appendices.)

#### Strengths of mobile applications for workplace health promotion

Generally, the studies mentioned many different advantages. Some were rather study-specific, but others were applicable to most WHP apps.

When deep-diving into factors that were found to enhance acceptance of WHP programmes positively when using mHealth, particularly the independence of time and place for use, accessibility and embedding into daily routines were mentioned as advantages most often [17, 31, 39, 46, 51]. These factors can be viewed as crucial, particularly for non-office workers who might work in shifts [31]. Of course, under the umbrella of global companies, accessibility anywhere and by anyone is also relevant. Furthermore, factors such as users’ curiosity (leading to use) [24, 52, 53, 58] and the advantage of supporting the user’s autonomy and self-guided learning were mentioned fairly often [9, 30, 35, 39, 41, 54]. Triggering users’ curiosity is important in enhancing first usage of the app, which might eventually lead to sticking with the programme, but also might trigger interest and widen the employee’s horizons, thereby influencing creativity [61]. Compared with ordinary on-site programmes, various authors in their investigations found ease of use and convenience to be advantageous factors [26, 27, 39]. Authors further hypothesised that perceived anonymity is an advantage that might reduce stigma compared with face-to-face interventions [31, 47]. Ideally, this would support individuals already suffering from health difficulties that they do not want to disclose at work among their colleagues. Particularly when talking about mental health difficulties, anonymity is a key element that employers wish to respect [47].

The results further indicate that one advantage that apps have over nontechnical interventions is the variety of functionalities they offer. In particular, authors highlighted reminders, prompts and alerts [9, 23, 24, 27, 38, 48, 53–55]. These functionalities strengthen context-aware triggers and behavioural change integration into daily life, which are viewed as two of the BCTs.

Several studies also identified self-monitoring functionalities [32, 37, 46, 47, 52–55], automatic data gathering and the possibility of personalization as advantages that increased app-based interventions’ effectiveness [9, 32, 35, 39, 42, 47, 48, 51, 53, 54]. Authors and participants in the studies also identified progress-tracking in real time, as well as real-time feedback, as advantages of WHP apps compared with ordinary WHP programmes [24, 28, 29, 33, 38, 49, 54, 56], again playing into personalization. Robroek et al. (2021) also mentioned tailored interventions as one major aim for future WHP interventions [7].

From an employer perspective, the most commonly mentioned positive factors of mHealth programmes, when compared with nontechnical interventions, were the ability to reach many employees due to smartphones’ wide distribution––possibly even reaching workers with nonstandard working hours, e.g., shift workers [9, 17, 26, 31, 34, 38, 39, 44, 49, 51, 53], as already mentioned under the factors of independence of time and place. Furthermore, authors referred to flexibility, the possibility of integrating various programmes into one app and cost-effectiveness [9, 23, 24, 26, 27, 32, 35, 38–41, 43, 44, 47, 56, 57]. Interestingly, one paper concluded further that employees found it beneficial that employers could monitor the workplace through apps and track activities because they finally could witness firsthand the difficulties that employees face [52]. This is clearly also an advantage from the employer’s perspective, as focus areas for complementing interventions can be identified using data from the apps. Furthermore, two studies concluded that infection risks also were reduced, which was essential during the COVID-19 pandemic [32, 39].

**Opportunities for** using mobile applications for workplace **health promotion.** Opportunities for using mHealth apps for WHP are defined as future options for the developments mentioned in the studies, such as those associated with embedding apps into other employee support package features and future technological developments. In particular, these apps’ projected cost-effectiveness compared with nontechnical solutions, the possibility of integrating app use into other WHP programmes, additional functionalities and evidence-based developments also were mentioned [24, 29, 31, 38, 39, 53, 55–57]. Clearly, these opportunities resonate with technical developments reported in the various studies over time. Including more functionalities and more WHP programmes into one app further indicates that current programmes might be pilot studies and that employers are only slowly starting to use the apps.

Moreover, these results reveal that there is room for further studies. In particular, the aspect of cost-effectiveness needs more in-depth research in terms of return on investment in improved health outcomes.

#### Weaknesses of mobile applications for workplace health promotion

Generally, the studies reported fewer disadvantages than advantages. Problems encountered regarding the use and acceptance of apps included the following: employees reporting a lack of time to use the apps, particularly at work–which also often is reported with regard to nontechnical solutions for WHP [9, 23, 24, 29, 39, 43, 44, 46, 53–55, 57, 58], and a lack of adherence and a drop in engagement over time [24, 25, 29, 30, 35, 38, 39, 42, 53]. Some authors pointed out disruptions in app use due to technical complications, e.g., a lack of connectedness between apps and other technologies, network issues or battery consumption woes [9, 25, 43, 48, 49, 53]. However, such technical difficulties certainly can be remedied and already might have decreased due to technical advances. Ahtinen et al. (2013) also reported that participants mentioned an inability to install an app on their private phones as a weakness, representing a discussion as to whether to use a private phone (and also cover private activities on the WHP app) or company phone–if provided–to install such an app. Furthermore, four authors stated that reminders are perceived as annoying or frustrating [9, 24, 41, 53]. Finally, app use at work could be perceived as inappropriate [24].

Apart from technical and usability issues, privacy concerns, anonymity and security issues also were raised [9, 38, 48, 55, 56]. Past authors already discussed the topic of privacy perception and indicated differences in data element perceptions of private and WHP health apps, of which developers and employers should be conscious [62].

Users also value making connections with others through physical personal contact, which is not a feature that apps provide [25, 55]. Surely, this is particularly critical for mental health challenges, i.e., giving the right advice and ensuring that apps do no harm. Finally, effectiveness and feasibility of support in severe medical or psychological cases were questioned [33, 43, 56].

#### Threats that may affect mobile application use for workplace health promotion

In examining future and potential pitfalls, a lack of scientific research was mentioned in particular. Furthermore, potential developments, e.g., such technological interventions’ sustainability, were questioned, particularly because engagement dropped fast. Individual studies also mentioned factors such as data privacy and anonymity, but also organizations’ influence as potential threats.

A list of mentioned factors can be found in Table 3.

#### The results’ generalizability

Of course, the results need to be put into the context of their generalizability. Due to the variety of methods used, the studies are not comparable as such. Notably, mixed-methods design studies and qualitative studies in general revealed more advantages and disadvantages due to close personal contact with participants. This should be kept in mind when interpreting the results.

Furthermore, studies published earlier included less technically advanced apps, including other challenges and opportunities. Generally, fewer disadvantages were found than advantages, potentially suggesting a bias towards positive reporting of study results.

However, some advantages might be disadvantages under certain conditions and vice versa. As Greenfield et al. (2016) stated, tracking working conditions was viewed as beneficial for employers, who could monitor workplaces, but viewed as violating privacy in other contexts, e.g., with regard to mental health topics [52]. Self-monitoring is another point. It is beneficial if applied correctly, but the lack of direct feedback and guidance by an expert might be risky with regard to some topics [25], as misinterpretation of results or misguided advice might be harmful to users’ mental health.

This scoping review’s results provide the first overview of potential advantages and disadvantages, but companies need to consider their own settings carefully while keeping all these factors in mind.

## Discussion

As described above, participation rates in ordinary WHP programmes often are low, and various factors have been proposed to account for this. To enhance participation rates and, thus, strengthen WHP and its effectiveness, new interventions and methods are necessary. As mobile apps are viewed as effective in various other areas of health care, it can be argued that they also may be effective for WHP [10]. However, knowledge on these apps’ potential is lacking, particularly in regard to testing apps in practical settings and using objective data, and health apps generally are not evidence-based [63]. This review was conducted to close this knowledge gap. As such, it aimed to evaluate the advantages and disadvantages associated with mobile apps compared with nontechnical WHP programmes based on a SWOT analysis (for a summary, see Table 3), as well as to provide an initial overview of their potential role in making decisions within companies and reveal their future research potential for academia.

The evaluated studies indicate that mobile apps (as a new means of supporting interventions) seem to be effective and beneficial under certain conditions, and specifically that they might be capable of integrating different areas of WHP, e.g., physical and mental well-being programmes, into one comprehensive programme. Still, questions remain about how they can be combined with other WHP programme aspects, particularly given that studies have emphasised the current lack of evidence-based mHealth interventions [35, 64].

Thus, WHP’s context and general approach need to be considered carefully to overcome disadvantages.

In the next section, we discuss our findings alongside the literature that has reported on the advantages and disadvantages of nontechnical WHP programmes.

### Reasons for using mobile applications for workplace health promotion

As described above, various strengths from using WHP apps were reported in the included studies. Effectiveness in terms of many health outcomes, convenience for the end-user, flexibility and cost-effectiveness were mentioned mainly as arguments for using these technologies for WHP. However, how beneficial are these apps potentially compared with nontechnical supported WHP programmes? The literature reports various challenges that explain low participation in non-app-based WHP offerings.

One aspect is that those with previous positive health behaviours are more likely to participate compared with non-active users [65, 66], thereby limiting effectiveness and contradicting WHP’s main preventive aim. Considering our results, some apps’ strengths might overcome this barrier. One study by van Drongelen et al. found that groups of people of various ages and demographic factors used its mobile app [67], and other studies examined apps and found they had a wide reach. Similar results were reported in a study on a web-based stress intervention, which found no differences among baseline stress levels, implying that those with health issues also participated [68]. One reason for this heterogeneity might be the perceived anonymity and nonstigmatisation associated with mHealth apps [31, 47].

Another factor limiting participation in WHP interventions in the past is related to obstacles perceived by users, e.g., time barriers, the perception of an overlap between work and home, and the perception that WHP is provided in inconvenient places [65, 69, 70].

The barrier of lack of time is, of course, difficult to solve. Even though this also was reported as a weakness in WHP apps, the possibility of usage anywhere and anytime, as well as personalised content, limited this barrier because of the flexibility provided to users.

This flexibility, due to 24-hour availability and independent of time and place, was appreciated by the participants in a study on truck drivers, who typically are difficult to reach in terms of local health promotion measures [34, 39, 52]. Of course, office workers also perceived this flexibility as positive [27, 38, 40, 47]. This was particularly the case because new ways of working experienced a boost during the COVID-19 pandemic, leading to even more pressure on employers to experiment with new measures for WHP tailored to employees’ needs [71]. Thus, apps can enable employers to reach a larger target group than is possible through nontechnical WHP interventions [9, 26, 31, 51], including shift workers [31, 33, 36, 57]. As most people bring their phones with them to work, with no extra device needed, this means less effort is needed [46].

Furthermore, app users and employers appreciate the possibility of adjusting and personalising mobile apps to suit a user’s preferences and needs, as well as workplace requirements [9, 32, 35, 42, 47, 48, 51, 53, 54]–a reported desire of users of nontechnical WHP interventions [69, 72]. Therefore, apps also should integrate different WHP programme parts [9, 54]. Mobile apps may even include certain private activities, e.g., personal jogging programmes, which can be effective, as employees frequently prefer to choose their own activities [65, 70, 73]. Thus, if employees do not want their professional and private lives to intermingle, this is not necessary. By including the option to personalise their own content, it may be possible to improve interest and motivation among users and limit dropout rates and failure to participate due to lack of interest [38, 69, 74–76]. Particularly due to the user’s desire to be autonomous and track their own status [9, 30, 35], self-guided learning and direct feedback [29, 33, 56]–together with integrated functionalities, e.g., setting reminders and incentives–can be used to improve long-term adherence to WHP [9, 23, 24, 27, 38, 48, 53–55].

Visualisation and self-monitoring, which often go hand-in-hand, also were identified as advantages in the literature on technology use in WHP [9, 33, 38, 47, 48, 53, 54, 56]. Showing users their achievements and progress through graphical feedback tailored to their own needs can increase users’ motivation. Visualisations and appealing interfaces also can make apps easier to use, making health improvement a positive experience and reinforcing users’ motivation [55]. Social collaboration and competition among colleagues are also highly valued features of mobile apps [40, 56]. Social support from colleagues [66, 69, 74, 77, 78] and supervisors and group pressure [72] also were mentioned as advantages of nontechnical interventions, and also were valued in the apps [24]. However, some authors cited social pressure as one weakness of technical solutions [24, 41], while others cited the lack of direct interaction as another [25, 55].

Nöhammer (2022) et al. summarised the preferences that users generally have in terms of WHP as the following: ‘personal benefit; social aspects, information; uncomplicated use; security and autonomy, plus time and participation in offer’. Uncomplicated use, personal benefit and information were rated as the most important by their study group. If developed in an attractive manner, those three aspects can be covered by apps, with uncomplicated use and information particularly easy to implement. Surely, personal benefit depends on the content provided [8].

In addition to offering advantages for the user, adapting apps and their functionalities to meet employees’ individual needs can reduce the number of interventions that companies need to roll out [9, 30, 54]. Individuals’ ability to access different functions and programmes themselves also may result in cost-saving benefits. In some cases, a combination of online (web- or app-based) and offline (on-site) services also might be feasible [79] because medical and psychological support are difficult to provide virtually. Advantages for employers that were mentioned included limited infection risk (particularly during the COVID-19 pandemic), as well as employers’ ability to assess working conditions objectively [32, 52].

The often-described positive effects from apps go beyond general effectiveness in terms of health outcomes and also can include factors such as engagement by arousing employees’ curiosity and feelings of connectedness to colleagues, but these factors were not studied.

### Arguments against using mobile applications for workplace health promotion

Although the evaluated studies identified several strengths of mHealth apps for workplace health interventions, they also identified weaknesses. Many factors against using mHealth relate to problems and obstacles that do not exist with nontechnical interventions. For example, studies revealed that employees complained about technical difficulties, e.g., battery consumption issues [9, 25, 43, 48, 49, 53].

Furthermore, not all studies found higher participation rates in WHP programmes, and many reported high attrition rates. These apps’ privacy and data security also were viewed as potential pitfalls that have not been addressed properly [9, 52, 55, 56], though there has been discussion of potential privacy and security measures in mHealth [80, 81]. Some truck drivers viewed data collection and monitoring by their employers as a positive feature, as it meant that employers were finally able to see the truckers’ working conditions [52]. Nevertheless, Dunkl and Jimenez (2017) criticised the lack of quality assurance associated with mHealth apps because apps can be developed by anyone without medical expertise, even though an employer’s provision of a health app might indicate quality of information [16]. Several studies also questioned the accuracy of some measurements taken by phones and, thus, apps’ potential for objective and reliable measurements [9, 55, 56]. Of course, the competition with privately available health apps should not be neglected and might lead to high attrition or costs to keep up with trends, even though users are curious at first [9, 55].

Furthermore, justifications for nonparticipation and, thus, dropout were reported in studies on ordinary WHP programmes, as well as apps, with participants making comments such as, ‘I forgot to subscribe’ and ‘I forgot to use it’, or simply reporting a lack of time to use it (particularly at work) [9, 24, 29, 39, 43, 44, 46, 53–55, 57, 58]. For app-based interventions, some participants even reported that using a phone was viewed as inappropriate or unpractical at times, particularly when in the workplace [24]. Furthermore, reminders or notifications were perceived as annoying or frustrating, limiting longer-term use [9, 24, 41, 53].

According to the literature, some of the issues that hamper participation in workplace health interventions cannot be solved with an app and, therefore, must be mentioned in a discussion of WHP apps’ disadvantages. These factors include employees’ own perceptions of their needs or desired benefits from the app and, thus, a lack of motivation to use it [55, 58]. This can be reinforced through monitoring and graphical feedback, but users still must become active themselves. Furthermore, the app would still need to be promoted as a programme within a company [40].

Considering that many employees do not want their private and working lives to merge [70, 76], this issue might be resolved (at least to some extent) by providing the option to install the app on company phones with functions that can be used only at work. Some studies found that one focal area of WHP programmes should be employees’ preference for personal contact. When addressing psychological issues, a personal component is essential and cannot be replaced by technology [43, 47, 56]. Furthermore, one challenge with such an intervention is how to make the measures available to individuals without a suitable device [58].

Of course, the cost of development, ongoing adaptations of apps and further developments over time to keep up with apps on the market and technical advances need to be deemed costs over time [24, 38].

Finally, extant studies have revealed that WHP often concentrates on personal well-being rather than work culture and systems. Of course, these aspects fall outside the impact area of the WHP method [7]. Furthermore, continuous involvement by management is crucial for successful implementation [82].

To sum up, some previously reported barriers obviously can be resolved, though it may not be possible to resolve others, e.g., physical contact using a purely technical solution.

### Mobile applications’ future potential for workplace health promotion

Trends are particularly important in evaluating mHealth’s future potential for WHP. Aside from their capability for advanced functionalities, mobile apps provide opportunities to integrate different aspects of workplace health into a single comprehensive programme. When embedded into companies’ other support functions, they could offer an effective addition. As technological development and research continue, mobile apps may be able to provide support that people are not yet aware of. Certainly, these programmes’ scientific evaluations and quality assurance would need to be strengthened and guaranteed.

Of course, potential pitfalls, e.g., security and privacy risks, also may increase in the future and need to be addressed by technical developments, government regulations and scientific work. As with all workplace health interventions, regulations and cultural changes might inhibit development further.

### Implications for research and practice

Our scoping review focussed on combining literature and, thus, research with practice. By summarising the factors that speak for or against using mHealth for WHP, we provide a summary of potentially important factors to consider when deciding whether to use apps for WHP. Companies’ decision-makers can use our SWOT summary and rate the factors based on relevance for their settings and importance in terms of their company values to make informed decisions.

Clearly, our scoping review is just the starting point to summarise relevant factors. Researchers can use these factors to delve deeper into some crucial aspects, e.g., testing cost-effectiveness. Quantitative research could evaluate these aspects’ weight further from employees and employers’ perspectives, as well as those of developers of such apps, to develop guidelines that make them more evidence-based.

### Review limitations

This review was conducted to obtain an overview of the current literature on WHP apps. Due to the broad search approach and the resulting heterogeneity of the included literature, a scoping review, rather than a systematic literature review, was the chosen approach. Another limitation is that not all criteria from the PRISMA guidelines were followed. No *a priori* study protocol was available, inter-rater reliability was not evaluated and no structured critical or risk appraisal was conducted. However, due to the goal of summarising the advantages and disadvantages of WHP apps, a broad approach to the study was required.

When considering the evidence summarised in this scoping review, it is important to keep these limitations in mind. As this is a scoping review, the included articles’ quality varies. Considering that myriad outcomes were considered in these studies, we did not evaluate mHealth’s effectiveness in general (e.g., by using GRADE). Some studies considered the same interventions, which limited variation among the studies and apps reviewed, and highlighted the need for further evaluation. Cultural and economic variations also were not considered.

Furthermore, nearly all the included studies claimed to demonstrate mHealth’s effectiveness for WHP, leading to potential bias whereby the authors report more advantages in line with their desired results. In addition to these limitations, the review included studies that varied in their methods and outcomes, making an overall evaluation of WHP difficult.

## Conclusion

Taking all the findings into consideration, it can be concluded that mHealth provides many opportunities for WHP. Obviously, different workplace contexts are suitable for using mHealth for WHP and demonstrate effectiveness in measuring outcomes. However, to ensure their effectiveness, individual, structured assessments of workplace settings are required before implementing mHealth apps for WHP.

Furthermore, the literature clearly stated that in addition to strengthening personal well-being, work culture and systems also need to be considered to support employee well-being effectively, which cannot be changed by applying a new WHP method.

Still, compared with previously applied WHP methods, apps’ flexibility, personalisation possibilities and wide reach offer definite potential, i.e., many previously identified limitations of nontechnical interventions can be overcome, and their positive attributes (e.g., personalisation of intervention methods to suit individual employees and the independence of time and location) can be integrated into well-developed mobile apps to adapt to current and new ways of working. However, usage barriers (e.g., smartphone availability, bugs and a lack of personal contact) specific to this technology exist.

Certainly, trends should be considered when deciding whether to implement such an app in a workplace, e.g., future developments in mobile device technologies, security/privacy aspects and quality assurance.

Finally, this review could serve as a valuable starting point for future research on WHP apps and provide an overview of various factors for companies to consider and value based on their individual needs.

## Supporting information

S1 Appendix Details of the included studies from the literature review between 2007 and 2022 on mobile applications for workplace health promotion. (DOCX)

S2 Appendix Found strengths, weaknesses, opportunities and threats (1/2). (DOCX)

S3 Appendix Continued: Found strengths, weaknesses, opportunities and threats (2/2). (DOCX)

S1 Checklist Preferred Reporting Items for Systematic reviews and Meta-Analyses extension for Scoping Reviews (PRISMA-ScR) checklist. (DOCX)
