# Supplementary material for: Advantages and disadvantages of mobile applications for workplace health promotion: A scoping review
Source: PLoS One. 2024 Jan 2;19(1):e0296212. doi: 10.1371/journal.pone.0296212 (PMC10760718; doi:10.1371/journal.pone.0296212)
Supplement: S1 Appendix — (DOCX) [file pone.0296212.s001.docx]

S1 Appendix: Details of the included studies from the literature review between 2007 and 2022 on mobile applications for workplace health promotion

| **Authors (year). Journal. (Reference)** | **Methods / Country and study group (n)** | **Intervention/ App** | **Effectiveness** | **Strengths / Opportunities** | **Weaknesses/ Threats** |
| --- | --- | --- | --- | --- | --- |
| Ahtinen et al. (2013). JMIR Mhealth Uhealth [46] | Field study of one-month, in-depth interviews, log files and questionnaire (N = 15) 60% female University staff in Finland | App based on the Acceptance and commitment therapy (ACT) to improve mental health | Showed high effectiveness in stress and life satisfaction, but not for psychological flexibility (z=0.06, P=0.950) Pre-post assessment: stress ratings (z=3.00, P=0.003) satisfaction with life scores (z=2.32, P=0.02) | -       Wide distribution of smartphones in population* -       Integration into daily life -       Unobtrusive monitoring* -       Convenience * -       Interactive*  -       Personalization*  -       Short and easy exercises -       Freedom in terms of usage location -       Accessibility (easy to open) -       Integration with devices already owned **Opportunities** - scheduled programs, reminders - convincing content instead of gamification | -       Not installed on private phones -       Time issues **Threats**  - Still in its infancy* |
| Villani et al. (2013). Psychological Services [37] | RCT (between Subject design)  (N =30) Nurses in Italy All Female | Self-help stress management for oncology nurses supported by a mobile tool | Improvements for anxiety state, anxiety trait reduction and coping skills acquisition | -       Treatment fidelity* -       Accessibility* |  |
| Ly, Asplund & Andersson (2014). Internet interventions [17] | RCT (N =73) 58% male Middle managers mostly Swedish or American | App for stress management for middle managers based on ACT | Effectiveness was found for the perceived general health (d = 0.41), perceived stress (d= 0.50), but not for multifactor leadership scale | -       Cost-effective -       Time-efficiency -       Larger reach than other interventions* -       Wide distribution of smartphones* -       Integration into daily life  -       Accessibility |  |
| van Drongelen et al. (2014). Scandinavian Journal of Work, Environment & Health [36] | RCT (N= 502) Airplane pilots in the Netherlands  4.5% female | App to reduce fatigue among airplane pilots | Effective for long term usage, significant improvement in fatigue, sleep, nutrition, and physical activity; participation rate after 6 months rather low | -       Independent of time and place * -       Personalization * -       Wide distribution of smartphone in population* | -        Older population less experienced with apps * |
| Greenfield et al. (2016). BMC Public Health [52] | Semi-structured focus groups  (N =34) only male truck drivers in UK | mHealth intervention and wearable for truck driver to promote health | General interest in the app, ambivalent about privacy and risk of monitoring by employer | -       Users are curious  -       Simple, straightforward functions -       Monitoring of all behavior for prevention -       Confidence and reassurance by monitoring -       Employers can see work conditions | -       Privacy and data collection |
| Muuraiskangas et al. (2016). JMIR Ment Health [58] | Process and effect evaluation, interviews, questionnaire  (N = 27) Employees of 2 communication and IT companies in Finland 67% female | App and web-based version to target stress management and mental health, based on ACT | No effect found, low participation rate (8.1% of all employees took the app into use) | -       Easily accessible* -       Curiosity of employees -       Interest in digital interventions  -       Fun  -       Enjoyment in tackling everyday issues using an app | -       Lack of perceived benefits -       Lack of perceived need -       No suitable phone (all platform needs to be included) -       Time issues; Management need to assign time for usage and participation -       Integration into daily life |
| Baek et al. (2018). Psychiatry Investigation [47] | Field-test of 4 weeks with questionnaires  N = 68  Employees of three large companies in Korea 63% male | Automated stress management application: With the application, they monitored their stress level and life style factors | After the intervention, perceived stress level was significantly decreased (BEPSI-K score (Psychosocial instrument) pre. vs. post. 14.27 vs. 11.00, F=12.49, p=0.001 in 2013; 12.05 vs. 10.00, F=17.18, p<0.001). In 2014, depression symptom severity was also significantly decreased (CES-D score pre- vs. Post-, 17.66 vs. 11.95, F=9.76, p=0.004). The effects were more significant in females and in those <35 years | - Flexibility - Accessibility anywhere anytime - Personalization - Reduces Stigma - High penetration of smartphones - Self-Monitoring - Short and customized comments - Anonymity - Usage of Short duration possible * - High reach of younger and female workers | **Threat** - may not be suitable for clinical conditions |
| de Korte et al. (2018). JMIR mHealth and uHealth [9] | Case study: Qualitative data: interviews and focus groups (N=43) Predominantly men Netherlands? | “Brightr” app tailored at workers behavior e.g., mental resilience, sleep, physical activity, nutrition and shift work |  | -     Visualization  -       Ease of use -       Prompts -       Learnability  -       Increase awareness -       Personalization -       User autonomy -       Integration into other WHP programs -       Context-aware  -       Adaptability  -       Accuracy (positive and negative) -       Large reach  -       Self-monitoring -       Cost-effectiveness | -       Batterie consumption -       Privacy -       Time consuming -       Phone compatibility /Availability -       Unsuitable reminder times -       Preference of private apps -       System quality -       Technical difficulties: Bugs and being slow |
| Mistretta et al. (2018). J. Occup. Environ. Med [35] | RCT (N = 60) Nurses in the USA Predominantly female | 6-week program in 3 groups; Mindfulness-based resilience training (MRBT), resilience-based smartphone program or control group App provided data on sleep and emotions | The in-person MBRT group significantly reduced in stress with moderate effect sizes (ds. -0.59 to -0.62). Both the in-person MBRT and smartphone groups significantly increased perceived well-being; | -       Cost efficient  -       Accessibility * -       Exercise autonomy  -       Personalization to users and organizations | -       Little research on mindfulness apps or stress -       No long-term usage |
| Möltner, Leve, & Esch (2018). Gesundheitswesen [50] | Intervention study using questionnaires N Intervention = 146;  N Control = 160 German-speaking participants 69% female | Meditation app usage for 14 days; App includes explanatory videos and meditation courses and session ("7 Mind") | Significant effect sizes: between pre and post-test in the intervention group for mindfulness (p < 0.001, η2 = 0.14), work engagement (0.05, η2 = 0.02), job satisfaction (p < 0.05, η2 = 0.02), emotional exhaustion (p < 0.001, η2 = 0.06), emotional intelligence (p < 0.001, η2 = 0.05) and self-efficacy (p < 0.01, η2 = 0.04); significantly better results for the intervention group compared to the control group are found with medium effect sizes for mindfulness and emotional exhaustion and small effect sizes for the other measures | - Ease of use* - Independent of time and location* - Integration into daily professional context |  |
| Peters et al. (2018). JMIR Mental Health [54] | Exploratory qualitative study to design app  (N = 60) Employees of male-dominated industries in Australia (92% male) | No intervention: Workshops to derive feature ideas and artifact that should be included into a mental health app for male-dominated workplaces (State fire and rescue service and multinational freight transport company) |  | -       Self-tracking -       Self-assessment -       Personalization -       Interactive  -       Easy accessibility -       Link to services provided -       Notifications and reminder -       Progress tracking -       Visual feedback -       Offline accessibility -       Brevity | -       Less experience -       Less free time |
| Boerema, Van Velsen, Hermens (2019). BMJ health & care informatics [48] | Intervention study: log files, questionnaire, interview (N=15) - equal gender distribution University staff in Netherlands | mHealth intervention based on a context-aware activity coach that predicts physical activity. 1 Week intervention with aged 50+ office workers | Total sedentary time was not reduced as a result of using the intervention.  Participants reduced their total time spend in long sitting (more than 45 min) (p <0.05) | -       Insights into physical activity patterns /awareness -       graphical interface -       Personalization -       Prompts -       Self-monitoring | - Technical issues: Losing wireless connection |
| Bostock et al. (2019). Journal of occupational health psychology [27] | RCT over 16 weeks follow-up (N = 238) Employees of two UK companies (Pharma and high-tech) 60% Female | “Headspace” meditation app with 10 to 20 min. guided audio meditations  8- week intervention with follow-up survey after 16 weeks | Significant improvements for well-being, distress, job strain, perceptions of workplace social support in intervention group compared to control group. Sustained positive effects after 16 weeks in well-being and job strain | -       popular method* -       Standardized instruction and objective measure of adherence in studies* -       Reminders -       Objective measures -       Convenient -       Cost-effective -       Flexibility | - lack of research |
| Coelhoso et al. (2019). Journal of medical Internet research [29] | 2 arm RCT in Brazil; employees of a private hospital (N=490) Baseline, midintervention (4 weeks after baseline), and postintervention (8 weeks after baseline) questionnaires in the app All women | (1) well-being mobile app based on relaxation, breathing, meditation, and positive psychology principles and  (2) control app, containing only instructions to self-observation for 20 min and recording of subjective levels of stress and wellbeing. | Both groups showed a significant increase in general well-being over time (F =5.27; P=.006) Significant increase in work-related well-being Only the intervention group (F =8.92; P<.001), and significant reduction in work-related and overall stress (F =5.50; P=.004 and F =8.59; P<.001) | - interaction with participants in context (in daily life) - Ecological momentary intervention - versatile, multifaceted and interactive - ubiquitous use of smartphones provides a widespread and effective way to promote change - instantly accessible  - more rigorous - objective measurement of adherence - accessibility* - preventive use* - reduces stigma* - self-monitoring in stressful situations* - Various functionalities e.g., Gamification, triggers, reminders * - real-time engagement* - simple, enjoyable, intuitive and interactive design **Opportunities** - cost-effectiveness - personalization - interactive support | - high attrition rates* - not easy to make time for exercises - difficult to find personal space to relax - one size fits all approach **Threats** - without systematic research potential harmful or not effective - lack of experimental evidence* |
| Hwang & Jo (2019). International Journal of Environmental Research and Public Health [33] | RCT Experimental group  (N = 26) and control group (N = 30) Nurses in Korea (95% female) | App-based stress management program used for 4 weeks by nurses | Significant changes in the experimental group in Perceived stress scale (P= .035), occupational stress scale (P = 0.04) emotional labor (P= 0.027), well-being (P= 0.005) and self-efficacy (P= 0.025) No significant differences in depression and anxiety scales | -       High proportion of smartphone users * -       Accessibility * -       Convenience and usefulness *  -       Independent of space and time * -       Usability of various problems* -       Real time tracking and feedback -       Privacy protection possible * -       Ease of use -       User-friendly -       Fun | -       Little research *  -       Personal contact needed for psychological issues * -       High effort |
| Kekkonen et al. (2019). International Conference on Persuasive Technology [53] | Interview-based qualitative study after an RCT, based on app-log data  Thematic analysis N =29 micro-entrepreneurs Finland? | Recover! App was developed to support entrepreneurs to recover from work-related strain and stress based on Self-determination theory Goals in the app were set based on the Transtheoretical model | For some, concurrent usage of wearables or other applications led to discarding our application. Users thought that the application was relatively persuasive, but technical issues reduced its persuasiveness noticeably. When functioning properly, self-monitoring and reminders were found to be supportive for users to achieve their goals. Unobtrusiveness was found to increase the persuasiveness of reminders, while self-monitoring always seems to be dependent on the user’s personal needs. | - wide reach* - self-monitoring - persuasive features - curiosity leads to usage -supports to take time for exercise - personalization /Customization  - automatic sensor-based pedometer - reminders **Opportunities** - connection to wearables would be helpful - further development of reminders and customization of content | - technical difficulties - need for more help - short usage - lack of time for usage at work - behavioral changes takes a lot of time - expectations based on previous experience not fulfilled  - additional stressor at work - misconception by users - reminders might be annoying - heterogenous target group makes development difficult |
| Weber, Lorenz & Hemmings (2019). Frontiers in psychology [38] | RCT with 6 weeks follow-up N =301 completed all questionnaires - predominantly women 6 European companies | “Kelaa” aims to reduce stress and increase wellbeing of the user, specifically in the workplace. Users learn new behaviors and best practices through different means, for example, based on CBT and mindfulness based cognitive therapy. The app is designed to implement lifestyle changes through (1) measuring behavior, cognitions, and emotions (tracking module) and (2) providing psychoeducational content (intervention module). Within the tracking module, users can track their stress, wellbeing, and resilience via short in-app questionnaires using validated scientific measures. The app also uses inbuilt sensors in smartphones (e.g., the accelerometer) to provide the opportunity to measure and track their sleep quality and quantity. | Using the mobile health intervention (vs. waitlist control group) significantly improved stress and wellbeing over time. Higher engagement in the intervention increased the beneficial effects. Additionally, use of the sleep tracking function led to an improvement in sleeping troubles. The intervention had no effects on measures of physical health or social community at work. | - integration into daily life * - easy accessible* - Scalable* - cost-effective* - personalized feedback - real-time tracking* - proactive and preventive approach for employers * - personalization opportunities* - anonymity* - control over progress pace* - link to daily routines* - targeted to workplace needs* - wide reach by broad scope - flexibility* -feeling of privacy* - independent of time and place* - individual choice of content - reminders and push notifications - may create a group feeling * - self-monitoring - may also be beneficial for colleagues due to cultural change **Opportunities** - growing number of apps and research | - data security needs to be ensured* - high rates of non-adherence  **Threats** - company communication is crucial - few apps are scientifically validated* |
| Billmann, Böhm & Krcmar (2020). Health Policy and Technology [44] | Case study; 3 months follow-up; log data and questionnaires  (N = 197) office workers in Germany 60% male | Evaluation of a objective health screening with app usage (app used to show results and provide information and challenges) | Amongst the two gender groups, significant differences were found in the overall number of actions performed (p = 0.004), viewing of the biomarkers ( p = 0.006) and health-promoting actions ( p = 0.003). Women performed significantly fewer actions overall (221.4 actions) than men (298.4 actions). In addition, men used the app for longer (35.4 days) compared to women (27.3 days, p = 0.023). In particular, men were viewing their biomarkers (p = 0.044) and their comparison ( p = 0.035) more often | -       independent of time and location* -       24h reachability*  -       Reach large audience* -       Self-monitoring* -       Adaptability*  -       Cost-effectiveness* -       Integration into daily life* -       High engagement | -       lack of knowledge on adoption* -       Lack of time |
| Bort-Roig et al. (2020). International journal of environmental research and public health [49] | Intervention study over 13 weeks with desk-based employees of 4 hospitals in Spain N = 141 82% female | TheW'@W-App, installed on the participants´ own smartphones, provided real-time feedback for occupational sitting, standing, and stepping, and gave access to automated strategies to sit less and move more at work. Changes between groups were assessed for total sitting time, sedentary bouts and breaks, and light and moderate-to-vigorous PA (activPAL3TM; min/day) between the baseline and after program completion. | Compared to the baseline, the W@W-App participants moved more at work (+46 min stepping, p = 0.039), reporting small but statistically significant changes in occupational light intensity activity (+2.4 min, p = 0.031) Compared to the active comparison group employees that used the W@W-App program increased their number of daily breaks and the time spent on short sedentary bouts (<20 min, p = 0.047) during weekends. Changes in shortest sedentary bouts (5–10 min) during weekends were also statistically significant (p < 0.05). No changes in workday PA or sitting were observed | - widely accessible* - self-monitoring* - real-time feedback - low-costs* - possibility to utilize motivational messages | technical issues (e.g., battery) |
| Emerson, Heavin & Power (2020). Proceedings of the 53rd Hawaii International Conference on System Sciences [56] | Mixed Method design: Exploratory, empirical case study in Ireland among healthcare workers   incl. App data, questionnaires and interviews N =70 app users N= 6 interviews Predominantly women | The app features included automatic recording of steps/activity using the phones in-built pedometer, an option to input personal data such as height/ weight, a suite of automated motivational messaging about the Steps to Health Challenge, and a feature to record level of wellness (mood) at work | The majority 85.7 (± 5.4 %) of participants strongly agreed or agreed that they found the app easy to use and 78.5 (± 6.3 %) strongly agreed or agreed that they liked the look and feel of the app (n=37). | - reliable tracking - Quantified self - socialization and competition - sharing of personal progress - simplicity* - integration into daily life* - clear feedback on progress* - low-costs* - immediate support* - easy to use - self-monitoring - real-time feedback - competition  - common topic at work **Opportunities** - future technological developments | - little evidence on effectiveness - privacy and data concerns - might not be right for severe cases - forgot to take phone for a walk - issue of accuracy  - gender differences - fear of consequences at work |
| Haque, Kangas, & Jämsä (2020). JMIR formative research [55] | Mixed methods design (Quantitative and qualitative) 4-week app usage among office workers compared to paper-based intervention Design, development and evaluation of the app (N=84) App usage, interviews, questionnaire Employees of 4 companies in 4 countries (62% male) | Physical activity app based on SDTs using goal setting, walking tracking and gamification 3 basic SDT psychological needs: increasing their levels of autonomy (ability to choose a daily walking task to reach a 10-minute goal after breakfast and lunch breaks), competence (feeling effective in their ongoing interactions with the social environment and to reach the daily walking goals), and relatedness (feeling connected with colleagues for the purpose of PA). | Compliance with app usage relatively low (experimental group, n=20; control group n=7).  mHealth app helped to increase PA compared to a paper diary (P=.033).  App supported 2 of the basic SDT psychological needs, namely autonomy (P=.004) and competence (P=.014), but not the needs of relatedness (P=.535). | - reminders - overcome paper-based barriers e.g., forgot to complete diary - automated recording of walking   **Opportunities** - cultural differences - more features and options could be added | - lack of time - holidays - laziness  - personal issues - disliked appearance  - already uses another mHealth app  - did not feel the need for this type of service - were already taking care of themselves - busy with their working lives - younger age and employment status correlated with discontinuation - lack of actual daily interaction with colleagues - privacy issues - inaccurate measurements  **Threats** - weather conditions influences outdoor PA - secure encryption and two factor authentication |
| Mascaro et al. (2020). Journal of Wellness [34] | longitudinal and randomized controlled design nonwhite call center employees in the US 1-year subscription N = 95 (89% Female) | Participants were randomized to either 6 weeks of daily mindfulness practice delivered by the Headspace app or to an open relaxation group that was instructed to relax any way they would like. Participants were instructed to complete the 10-minute version of levels 1, 2, and 3 of the Foundation series during their 12-minute break each day. | Employee C-reactive protein levels were positively correlated with subsequent meditation practice time (r(22) = 0.60, p = 0.002).. Employees who reported wanting to use the app to manage stress were most likely to use it, and women practiced significantly more than men. No other psychological resources were significantly correlated with practice time. Employees randomized to mindfulness had a significant increase in self-reported mindfulness scores, but did not have significant improvements in any other psychological or performance domains | - for rural and blue-collar workers apps have better access * - reduce health disparities* - remove the time barrier of ordinary programs |  |
| Morris et al. (2020). International journal of environmental research and public health [23] | A 3- arm quasi-randomized intervention over 12 weeks N = 34 at 12 weeks; predominantly female (64%) UK | Prompt for standing up at 30 or 60 minutes Throughout the trial, intervention participants received pop-up notifications which stated ‘time to stand up’, accompanied by a sound and/or a vibration alert, on their smartphone at the pre-selected break frequency. To log a break, participants manually clicked the pop-up notification. | The Prompt-60 arm was associated with a reduction in occupational sitting time at 6 weeks ('-46.8 min/8 h workday [95% confidence interval = '-86.4, '-6.6], p < 0.05) and 12 weeks ('-69.6 min/8 h workday ['-111.0, '-28.2], p < 0.05) relative to the No-Prompt Comparison arm. Sitting was primarily replaced with standing in both arms (p > 0.05). Both groups reduced time in prolonged sitting at 12 weeks (Prompt-30: '-27.0 [􀀀99.0, 45.0]; Prompt-60: '-25.8 ['-98.4, 47.4] min/8 h workday; both p > 0.05). There were no changes in steps or cardiometabolic risk. | - multicomponent intervention - low costs* - widely accessible* -portable * - large usage among working-population* - ability to set prompts and reminders **Opportunities** - effectiveness relies on participant engagement |  |
| Chan et al. (2021). TMS Proceedings 2021 [28] | two-arm RCT for 4 week intervention N = 205 working adults in China  Gender distribution not stated | Clara consisted of more than 700 psycho-education sessions and evidence-based exercises related to cognitive behavioral and mindfulness principles. | Significant time (before vs. after intervention) by group (intervention vs. control) interaction effect was found, F (1, 175) = 56.67, p < .001, η²= .25. Participants in the intervention group reported less depression, anxiety, and stress symptoms after the intervention (M = 14.38, SD = 15.14) than before the intervention (M = 28.90, SD = 17.99). The attrition rate was 86%. | - less stigma* - personalization* - consumer-friendly* - real-time monitoring* - intervention in daily life* |  |
| Lu et al. (2021). Frontiers in Psychology [45] | Field study of 8- week program N = 218  various countries e.g., Australia, India, Indonesia, USA Equal gender distribution: Women: 50.5% | Awakened Mind: 8- week mindfulness program with 4 modules: Module 1: introductory program to mindfulness meditation, breathing, and relaxation techniques. Module 2: fundamentals of mindfulness and mindlessness and concept of acceptance. Module 3: attentional awareness and body scanning exercises. Module 4: mindfulness and how the techniques of mindfulness could be adapted to overcome potential workplace challenges | Mindfulness increased over time (γ = 0.14, p < 0.01), and time also had indirect effects on emotional exhaustion, work engagement, and job satisfaction, through mindfulness. Supplementary growth curve analyses on the improvement of mindfulness over time showed a slight decrease in the positive effect of time on mindfulness. |  |  |
| Morris et al. (2021). International journal of environmental research and public health [24] | A three-arm quasi-randomized with 12 week follow-up N = 44 Two focus groups N = 8 UK Predominantly female (64%) | intervention included prompts at 30 or 60 min intervals delivered via a smartphone application, and a no-prompt comparison arm. | Contextual findings indicate that when working on a sedentary task (i.e., reading or screen-based work) and located at an individual workstation, hourly prompts may be more acceptable and feasible for promoting a reduction in total and prolonged sedentary time compared to 30 min prompts. Interpersonal support also appears important for promoting subtle shifts in sedentary working practices | - real time tracking*  - across a variety of settings* - prompts, cues and self-monitoring* - high proportion of population has smartphone* - Cost-effective - widely accessible  - users are curious - interpersonal support  - cultural factors - peer support **Opportunities** - linkage to other technologies to improve automatic tracking  - additional functionalities as gamification, challenges, messages or organizational engagement  - refinement based on feedback possible | - user engagement drops over time* - lack of time to participate due to high workload - not always practical, feasible or appropriate to carry the phone - prompts caused disruption to concentration - enhanced feeling of stress - perceived interpersonal expectations  - adherence dropped over time - manual entry was perceived as clunky and inconvenient  **Threats** - limited sustainability of an e-health smartphone intervention |
| Rich, Ogden & Morison (2021). International Journal of Workplace Health Management [40] | Randomized waitlist control trial (2 months) among UK university employees using questionnaires N = 101 incl. Post program questionnaire Predominantly female (69.9%) | Headspace app Headspace® was the mindfulness-based self-help training application (app) used for this study. Headspace® consists of 30 foundation sessions of 10 minutes each which are available for individuals to use at their convenience. All sessions are repeatable. Headspace® uses audio, video, animations and exercises which incorporate opportunities for breath awareness, body scans, focus, and motivation and intentions | significant improvements in aspects of mindfulness relating to acting with awareness (d= .51, medium effect size), nonreactivity (d= .39, small effect size) and the total mindfulness score (d= .50, medium effect size) for those offered Headspace®. No significant differences were found for the describing, observing and nonjudgement aspects of mindfulness. Participants offered the MBP showed a significant reduction in perceived stress (d= -.39, small effect size). No significant differences were found for any of the work-related measures. | - shorter sessions - usage at users time availability - flexibility - reminder* - community sharing* - low costs* | - input from the employer is needed to encourage participation **Threats** - organizational measures are also necessary to complement and app and be effective against stress |
| Sasaki et al. (2021). Journal of Medical Internet Research [41] | Three-arm RCT among nurses in Vietnam N =949  6 week program and 7 months follow-up Predominantly female (84,9%) | The intervention groups were a 6-week, 6-lesson program offering basic cognitive behavioral therapy (CBT-based stress management skills), provided in either free-choice (program A) or fixed order (program B). Work engagement was assessed at baseline and 3-month and 7-month follow-ups in each of the 3 groups | The scores of work engagement in both intervention groups improved from baseline to 3-month follow-up, and then decreased at the 7-month follow-up, while the score steadily increased from baseline to 7-month follow-up in the control group. Program B showed a significant intervention effect on improving work engagement at the 3-month follow-up (P=.049) with a small effect size (Cohen d= 0.16; 95% CI 0.001 to 0.43]). Program A showed nonsignificant trend (d=0.13; 95% CI –0.014 to 0.41; P=.07) toward improved engagement at 3 months. Neither program achieved effectiveness at the 7-month follow-up. | - wide reach in low and middle income countries* - personalization * - fully automated - self-guided - accessibility - low cost - little experts guidance needed - high completion rate **Opportunities** - refresher sessions might be needed | - usage dependent on culture and smartphone literacy - lack of intensity and repeatability for long-term effectiveness - employees need to make choices in the app - social pressure - too many reminders caused frustration |
| Bartlett et al. (2022). JMIR mHealth and uHealth [25] | RCT among Tasmanian State employees with 6 months follow-up  N = 211 72,5% female | The app includes lessons, activities, and guided meditations, and is supported by 4 instructional emails delivered over 8 weeks. Engagement with the app for 10-20 minutes, 5 days a week, was recommended. Those were supported by on-site workshops (among one group) | No effect for app only group: Compared with the waitlist control group at T1, no significant change in perceived stress was observed in either active group. However, the app+classes group reported lower psychological distress (β=−1.77, SE 0.75; P=.02; Cohen d=–0.21) and higher mindfulness (β=.31, SE 0.12; P=.01; Cohen d=0.19). These effects were retained in the app+classes group at 6 months. No significant changes were observed for the app-only group or for other outcomes. There were no significant changes in observer measures at T1, but by time point 2, the app+classes participants were more noticeably mindful and altruistic at work than app-only participants | - popular and accessible* - self-guided learning* - accurate measure of program engagement * - less time issues compared to group with onsite workshops  - practice routine better established | - high attrition rate (85% - program with onsite workshops was perceived more beneficial and useful) - technical difficulties - onsite would have helped to motivate - absence of feedback and guidance by a teachers - no opportunity to discuss with others |
| Bonn et al. (2022). Journal of Medical Internet Research [26] | 3-armed parallel RCT (one control group, one group with app only and one with additional telephone counseling) Follow-up at 3 months N = 191 of 4 companies 2 white collar and 2 blue-collar workers companies in Sweden 61,5% of participants male | Results from the health profile were discussed with the health coach at the baseline meeting. The participant and the health coach decided on which lifestyle behavior to target based on the health profile. Thereby, each participant received a personalized intervention, customized based on their needs and goals. The intervention could target any of the following 6 areas: diet, physical activity, sleeping habits, stress, alcohol, or tobacco use. | Participants receiving only the app had statistically significantly lower BMI ( –0.35, 95% CI –0.61 to –0.09), body weight (1.08, 95% CI –1.92 to –0.26), waist circumference (–1.35, 95% CI –2.24 to –0.45), and body fat percentage (–0.83, 95% CI –1.65 to –0.02) at follow-up compared to the controls. There was a statistically significant difference in systolic blood pressure between the two intervention groups at follow-up (3.74, 95% CI –7.32 to –0.16); no other statistically significant differences in outcome variables were seen. | - large-scale and cost effective - integration into daily life*  - Independent of SES - availability anytime and anywhere - convenience | - little evidence on effectiveness |
| Deady et al. (2022). Psychological Medicine [30] | RCT with follow-up at 5 weeks, 3 and 12 months  N = 2271 (predominantly male) Australia | Headgear, 30-day behavioral activation and mindfulness intervention | Intervention group had fewer depressive symptoms over the course of the trial compared to those assigned to the control (F3,734.7 = 2.98, p = 0.031). Prevalence of depression over the 12-month period was 8.0% and 3.5% for controls and HeadGear recipients, respectively, with odds of depression cases amongst the intervention group of 0.43 ( p= 0.001, 95% CI 0.26–0.70). | -functional and economic benefits  - effective to prevent depression cases - empowers individuals  - | - high attrition rate |
| Gnanapragasam et al. (2022). The British Journal of Psychiatry [31] | Multicenter parallel group RCT of healthcare workers (acute hospital providers and mental health providers) in UK 8-week follow-up N = 894 - predominantly women | The app seeks to promote behavior change and positive wellbeing habits, and is designed to promote mental well-being, manage stress and improve sleep.  The app included six focus areas to work on that users choose for (relaxation, sleep, anxious thoughts, feeling down, self-esteem, stress). The focus area influences the programs and activities in the app.  Examples of techniques are 4–7–8 breathing, progressive muscle relaxation, (CBT)-based cognitive restructuring, gratitude journaling, guided audios, desk exercises or physical activity program | Participants randomized to the app had a reduction in psychiatric morbidity symptoms (aMD = −1.39, 95% CI −2.05 to −0.74), improvement in well-being (aMD = 0⋅54, 95% CI 0⋅20 to 0⋅89) and reduction in insomnia (adjusted odds ratio (aOR) = 0⋅36, 95% CI 0⋅21 to 0⋅60). No other significant findings were found, or adverse events reported. | - scaled at pace - wide access - easy to access - time-flexible support - wide reach  -anonymity - feasible for shift workers **Opportunities** - need to be embedded in other employee support package parts | - app usage decreased overt time **Threats** - more studies needed |
| Hirshberg et al. (2022). Journal of Educational Psychology [32] | Pragmatic randomized wait-list controlled trial with 3 months follow-up N = 662 public school employees In the USA (predominantly female 88%) | a free 4-week smartphone-based meditation app designed to train key constituents of well-being (Healthy Minds Program [HMP]): Two introductory audio lessons and two introductory guided meditations (one sitting meditation and one active meditation), followed by one week of content for each pillar of wellbeing. Each week contains two brief podcast lessons (5 to 7 minutes each) with key insights and practical examples. In addition, each week contains three guided meditations related to the respective pillar. Participants can choose between active or sitting practices, and practice length (5 to 30 minutes). In total, the intervention contains 10 lessons and 14 guided meditations. | HMP predicted significantly larger reductions in psychological distress at post intervention (Cohen’s d =  .53, 95% CI [ .69,  .38], p , .001) and at the 3-month follow-up (d =  .33 [ .48,  .18], p , .001).  HMP was equally effective among participants with elevated baseline anxiety and depressive symptoms. These | - limits infections risk (e.g., during Covid) - accessed at scale  - low costs - easily modified and personalized |  |
| Huberty et al. (2022). JMIR mHealth and uHealth [43] | Pragmatic, cluster randomized controlled trial among employees of a large, multisite employer in USA over 8 weeks (Equal gender distribution) N = 192 completed all assessments | Calm App: includes meditation using mindfulness components, breathing techniques, and body scans Participants in the Calm intervention group were instructed to use the Calm app for 10 minutes per day for 8 weeks; individuals with elevated baseline insomnia symptoms could opt-in to 6 weeks of sleep coaching. All outcomes were assessed every 2 weeks, with the exception of medical visits (weeks 4 and 8 only). | Generally: small to medium effects Complete-case analysis at week 8: employees in intervention group experienced significant improvements in depression (P=.02), anxiety (P=.01), stress (P<.001), insomnia (P<.001), sleepiness (P<.001), resilience (P=.02), presenteeism (P=.01), overall work impairment (P=.004), and nonwork impairment (P<.001), and reduced medical care visit frequency (P<.001) and productivity impairment costs (P=.01), relative to the waitlist control.  Intent-to-treat analysis at week 8: Significant benefits of the intervention for depression (P=.046), anxiety (P=.01), insomnia (P<.001), sleepiness (P<.001), nonwork impairment (P=.04), and medical visit frequency (P<.001). | - Cost-effectiveness - viable and effective alternative to in-person approaches - feasible and scalable solution | - low download rate  - additional support might be needed to prevent mental health problems - lack of time to use the app - lack of motivation - technical difficulties - implementation difficulties |
| Nuijten et al. (2022). JMIR mHealth and uHe[42]alth | 2-arm randomized intervention trial 8- week program:  App data + questionnaires Employees of governmental organizations in Belgium N =176 predominantly female (72%) | 8-week digital health promotion campaign designed to promote walks, bike rides, and sports sessions. Participants could track their performance on two social leaderboards: one for their own performance and one for the organizational department. Newsfeed in the app for information Personalized treatment that tailored the complexity parameters based on participants’ self-reported capabilities and goals and a control treatment where the complexity parameters were set generically based on national guidelines. | The results indicated that engagement with the program inevitably dropped over time. However, engagement was higher for participants who had set themselves a goal in the intake survey. The impact of personalization was especially observed for frequency parameters because the personalization of sports session frequency did foster higher engagement levels, especially when participants set a goal to improve their capabilities. In addition, the personalization of suggested ride duration had a positive effect on self-perceived biking performance | - gamification options - personalization possibility (based on preferences and needs) - based on daily routines - user's autonomy* - tailored goal setting* | - engagement dropped over time - goal setting was used by little users - users in precontemplation or contemplation phase showed little engagement - self-reported data is not reliable - rewards are perceived unfair |
| Puzia et al. (2022).. Environmental and Occupational Health Practice [57] | cross-sectional survey among remote working employees in the USA N = 400 ; predominantly women (74%) | Calm App: The Calm app is a mobile and web-based app that offers guided meditations, breathing exercises, relaxation music with nature soundscapes, mindful sleep content, light stretching, and mindful movement exercises | 20% of remote workers used Calm at work.  Possibility to utilize the app at work was positively associated with team mindfulness (β = 2.39, p = 0.005) and psychological safety (β = 1.85, p = 0.005).  Using Calm with team members was significantly associated with team mindfulness (β = 2.99, p = 0.046) and discussing Calm with team members was significantly associated with psychological safety (β = 1.17, p = 0.029). | - on demand access anytime and anywhere* - easy access* - discrete * - cost-effective* **Opportunities** - employers should encourage usage by allocating specific times and discussion content on the app | - often no opportunity to use the app at work **Threats** - effectiveness is dependent on usage rate |
| Rick et al. (2022). International Conference on Digital Human Modeling and Applications in Health, Safety, Ergonomics and Risk Management [51] | App usage data and questionnaires  UK, USA, Canada, Australia N = 2946 working adults (Predominantly women: 64,8%) | "HeadUp" App  Tailored app based on private and work-related factors Offers ability to set goals | Employee well-being and usage behavior are significantly positively correlated (r = .158, p < .001, n = 2941), as well as well-being is also significantly positively correlated with psychosocial workplace characteristics (r = .296, p < .001, n = 2915). Both correlations show small effects Age correlated with all variables. Older used the app more often. Women used it more often Effect of psychosocial workplace characteristics on employee well-being was observed, with the effect being stronger for male employees (βm = .402, βf = .306; p < .001). | - widespread appeal - accessibility - ability to reach large populations - low costs - individual support for goals and motivation - simple  - continuously support - availability anytime and anywhere - personalization | - usage is gender specific |
| Xu et al. (2022).. Emergency medicine Australasia: EMA [39] | Two-arm RCT over 3 months in Australia Emergency department staff N =98 completed all questionnaires predominantly Women (78%) | The Headspace app is a commercially available app. Mindfulness meditation app | in the intention-to-treat analysis a statistically significant improvement of perceived stress levels (F = 15.70, P < 0.001), all three components of burnout (emotional exhaustion [F = 14.22, P < 0.001], depersonalization [F = 3.62, P = 0.030], personal accomplishment [F = 7.51, P < 0.001]), mindfulness (F = 8.83, P < 0.001) and wellbeing levels (F = 10.71, P < 0.001) from pre-intervention to 3 months later with small effect sizes was found. | - less time consuming - easy access - cost-effectiveness* - increased reach* - ideal for workers who cannot participate in face-to-face activities* - convenient stress management - self-help intervention  - social distancing possible - flexibility - accessibility anytime anywhere  - personalization - low costs  Opportunities - need to be embedded in other activities to support employees | - high drop out - high stress did not lead to usage - Difficult to implement into daily life of healthcare workers - lack of time or mental capacity to learn new skills |
