# Supplementary material for: Advantages and disadvantages of mobile applications for workplace health promotion: A scoping review
Source: PLoS One. 2024 Jan 2;19(1):e0296212. doi: 10.1371/journal.pone.0296212 (PMC10760718; doi:10.1371/journal.pone.0296212)
Supplement: S2 Appendix — (DOCX) [file pone.0296212.s002.docx]

S2 Appendix: Found Strengths, weaknesses, opportunities and threats (1/2)

|  | Total | Number of X^a^ | Number of Y^b^ | Ahtinen et al. (2013) | Villani et al. (2013) | Ly et al. (2014) | van Drongelen et al. (2014) | Greenfield et al. (2016) | Muuraiskangas et al. (2016) | Baek et al. (2018) | de Korte et al. (2018) | Mistretta et al. (2018) | Möltner et al. (2018) | Peters et al. (2018) | Boerema et al. (2019) | Bostock et al. (2019) | Coelhoso et al. (2019) | Hwang & Jo (2019) | Kekkonen et al. (2019) | Weber et al. (2019) |
| --- | --- | --- | --- | --- | --- | --- | --- | --- | --- | --- | --- | --- | --- | --- | --- | --- | --- | --- | --- | --- |
| **Strengths** |  |  |  |  |  |  |  |  |  |  |  |  |  |  |  |  |  |  |  |  |
| **Acceptance / Adherence / Use** |  |  |  |  |  |  |  |  |  |  |  |  |  |  |  |  |  |  |  |  |
| Independent of time and place | 13 | 7 | 6 | x |  |  | y |  |  | x |  |  | y |  |  |  |  | y |  | y |
| Portable | 1 | 0 | 1 |  |  |  |  |  |  |  |  |  |  |  |  |  |  |  |  |  |
| Embedded in daily routines and work life | 9 | 3 | 6 | y |  | x |  |  |  |  |  |  | x |  |  |  |  |  |  | y |
| Accessible | 16 | 8 | 8 | x | y | x |  |  | y |  |  | y |  |  |  |  | x | y |  | y |
| Easy to access | 3 | 3 | 0 |  |  |  |  |  |  |  |  |  |  | x |  |  |  |  |  |  |
| Offline access | 1 | 1 | 0 |  |  |  |  |  |  |  |  |  |  | x |  |  |  |  |  |  |
| Short and easy exercises | 3 | 3 | 0 | x |  |  |  |  |  | x |  |  |  |  |  |  |  |  |  |  |
| Short usage possible /Brevity | 2 | 1 | 1 |  |  |  |  |  |  | y |  |  |  | x |  |  |  |  |  |  |
| Supports taking time to exercise | 1 | 1 | 0 |  |  |  |  |  |  |  |  |  |  |  |  |  |  |  | x |  |
| Less time consuming compared to ordinary programs | 3 | 3 | 0 |  |  |  |  |  |  |  |  |  |  |  |  |  |  |  |  |  |
| Users are curious (leads to usage) | 4 | 4 | 0 |  |  |  |  | x | x |  |  |  |  |  |  |  |  |  | x |  |
| Self-guided (learning) | 4 | 3 | 1 |  |  |  |  |  |  |  |  |  |  | x |  |  |  |  |  |  |
| User's Autonomy | 4 | 3 | 1 |  |  |  |  |  |  |  | x | x |  |  |  |  |  |  |  |  |
| Control over progress pace | 1 | 0 | 1 |  |  |  |  |  |  |  |  |  |  |  |  |  |  |  |  | y |
| Popular technology | 2 | 0 | 2 |  |  |  |  |  |  |  |  |  |  |  |  | y |  |  |  |  |
| Interaction with participants in daily life | 2 | 1 | 1 |  |  |  |  |  |  |  |  |  |  |  |  |  | x |  |  |  |
| Common topic at work | 1 | 1 | 0 |  |  |  |  |  |  |  |  |  |  |  |  |  |  |  |  |  |
| Interpersonal/ peer support | 1 | 1 | 0 |  |  |  |  |  |  |  |  |  |  |  |  |  |  |  |  |  |
| Interactive | 1 | 0 | 1 | y |  |  |  |  |  |  |  |  |  |  |  |  |  |  |  |  |
| Reduces stigma | 3 | 1 | 2 |  |  |  |  |  |  | x |  |  |  |  |  |  | y |  |  |  |
| Anonymity | 3 | 2 | 1 |  |  |  |  |  |  | x |  |  |  |  |  |  |  |  |  | y |
| Privacy protection possible | 1 | 0 | 1 |  |  |  |  |  |  |  |  |  |  |  |  |  |  | y |  |  |
| Simple, enjoyable, intuitive and interactive design | 1 | 0 | 1 |  |  |  |  |  |  |  |  |  |  |  |  |  | y |  |  |  |
| Fun | 2 | 2 | 0 |  |  |  |  |  | x |  |  |  |  |  |  |  |  | x |  |  |
| Enjoyment in tackling everyday issues using an app | 1 | 1 | 0 |  |  |  |  |  | x |  |  |  |  |  |  |  |  |  |  |  |
| Convenient | 5 | 3 | 2 | y |  |  |  |  |  |  |  |  |  |  |  | x |  | y |  |  |
| Simplicity | 2 | 1 | 1 |  |  |  |  |  |  |  |  |  |  |  |  |  |  |  |  |  |
| Ease of use | 4 | 3 | 1 |  |  |  |  |  |  |  | x |  | y |  |  |  |  | x |  |  |
| User Friendly | 2 | 1 | 1 |  |  |  |  |  |  |  |  |  |  |  |  |  |  | x |  |  |
| Scaled at pace | 1 | 1 | 0 |  |  |  |  |  |  |  |  |  |  |  |  |  |  |  |  |  |
| Usability of various problems | 1 | 0 | 1 |  |  |  |  |  |  |  |  |  |  |  |  |  |  | y |  |  |
| Widespread appeal | 1 | 1 | 0 |  |  |  |  |  |  |  |  |  |  |  |  |  |  |  |  |  |
| Cultural factors | 1 | 1 | 0 |  |  |  |  |  |  |  |  |  |  |  |  |  |  |  |  |  |
| **Functionalities** |  |  |  |  |  |  |  |  |  |  |  |  |  |  |  |  |  |  |  |  |
| Persuasive Features | 1 | 1 | 0 |  |  |  |  |  |  |  |  |  |  |  |  |  |  |  | x |  |
| Simple, straightforward functions | 1 | 1 | 0 |  |  |  |  | x |  |  |  |  |  |  |  |  |  |  |  |  |
| Various functionalities e.g., gamification, reminders, triggers | 1 | 0 | 1 |  |  |  |  |  |  |  |  |  |  |  |  |  | y |  |  |  |
| Multi-component intervention | 1 | 1 | 0 |  |  |  |  |  |  |  |  |  |  |  |  |  |  |  |  |  |
| Reminders & Notifications | 10 | 9 | 1 |  |  |  |  |  |  |  | x |  |  | x | x | x |  |  | x | x |
| Gamification | 1 | 1 | 0 |  |  |  |  |  |  |  |  |  |  |  |  |  |  |  |  |  |
| Competition | 1 | 1 | 0 |  |  |  |  |  |  |  |  |  |  |  |  |  |  |  |  |  |
| Sharing of personal progress | 2 | 1 | 1 |  |  |  |  |  |  |  |  |  |  |  |  |  |  |  |  |  |
| Integration in other devices | 1 | 1 | 0 | x |  |  |  |  |  |  |  |  |  |  |  |  |  |  |  |  |
| Accuracy (Negative and positive) | 1 | 1 | 0 |  |  |  |  |  |  |  | x |  |  |  |  |  |  |  |  |  |
| Unobtrusive monitoring | 1 | 0 | 1 | y |  |  |  |  |  |  |  |  |  |  |  |  |  |  |  |  |
| Quantified self | 1 | 1 | 0 |  |  |  |  |  |  |  |  |  |  |  |  |  |  |  |  |  |
| Visualization | 1 | 1 | 0 |  |  |  |  |  |  |  | x |  |  |  |  |  |  |  |  |  |
| Learnability | 1 | 1 | 0 |  |  |  |  |  |  |  | x |  |  |  |  |  |  |  |  |  |
| 24 h reachability | 1 | 0 | 1 |  |  |  |  |  |  |  |  |  |  |  |  |  |  |  |  |  |
| **Effectiveness** |  |  |  |  |  |  |  |  |  |  |  |  |  |  |  |  |  |  |  |  |
| Personalization /Customization | 15 | 10 | 5 | y |  |  | y |  |  | x | x | x |  | x | x |  |  |  | x | y |
| Individual choice of content | 1 | 1 | 0 |  |  |  |  |  |  |  |  |  |  |  |  |  |  |  |  | x |
| Self-monitoring (also in stressful situations) | 12 | 8 | 4 |  |  |  |  |  |  | x | x |  |  | x | x |  | y | x | x | x |
| Automatic and reliable self-monitoring (e.g., sensor-based pedometer) | 6 | 6 | 0 |  |  |  |  | x |  |  |  |  |  |  |  | x |  |  | x |  |
| Progress tracking in real time | 4 | 1 | 3 |  |  |  |  |  |  |  |  |  |  | x |  |  |  |  |  | y |
| Confidence and reassurance by monitoring | 1 | 1 | 0 |  |  |  |  | x |  |  |  |  |  |  |  |  |  |  |  |  |
| Insights into physical activity patterns /awareness | 2 | 2 | 0 |  |  |  |  |  |  |  | x |  |  |  | x |  |  |  |  |  |
| Real-time feedback / Ecological momentary intervention | 4 | 3 | 1 |  |  |  |  |  |  |  |  |  |  |  |  |  | x | x |  |  |
| Visual feedback | 2 | 2 | 0 |  |  |  |  |  |  |  |  |  |  | x | x |  |  |  |  |  |
| Personalized feedback | 1 | 1 | 0 |  |  |  |  |  |  |  |  |  |  |  |  |  |  |  |  | x |
| Individual, tailored support for goals and motivation | 2 | 1 | 1 |  |  |  |  |  |  |  |  |  |  |  |  |  |  |  |  |  |
| Practice routines can be better established | 1 | 1 | 0 |  |  |  |  |  |  |  |  |  |  |  |  |  |  |  |  |  |
| Rigorous | 1 | 1 | 0 |  |  |  |  |  |  |  |  |  |  |  |  |  | x |  |  |  |
| Treatment fidelity | 1 | 0 | 1 |  | y |  |  |  |  |  |  |  |  |  |  |  |  |  |  |  |
| Effective to prevent depression cases | 1 | 1 | 0 |  |  |  |  |  |  |  |  |  |  |  |  |  |  |  |  |  |
| Immediate support | 2 | 1 | 1 |  |  |  |  |  |  |  |  |  |  |  |  |  |  |  |  |  |
| High completion rate | 1 | 1 | 0 |  |  |  |  |  |  |  |  |  |  |  |  |  |  |  |  |  |
| High engagement | 1 | 0 | 1 |  |  |  |  |  |  |  |  |  |  |  |  |  |  |  |  |  |
| Overcomes barriers of ordinary programs e.g., forgot to complete diary | 1 | 1 | 0 |  |  |  |  |  |  |  |  |  |  |  |  |  |  |  |  |  |
| Time efficient | 1 | 1 | 0 |  |  | x |  |  |  |  |  |  |  |  |  |  |  |  |  |  |
| **Employer perspective** |  |  |  |  |  |  |  |  |  |  |  |  |  |  |  |  |  |  |  |  |
| Customization to organization | 1 | 1 | 0 |  |  |  |  |  |  |  |  | x |  |  |  |  |  |  |  |  |
| Adaptability | 4 | 2 | 2 |  |  |  |  |  |  |  | x |  |  |  |  |  |  |  |  | y |
| Flexibility | 5 | 4 | 1 |  |  |  |  |  |  | x |  |  |  |  |  | x |  |  |  | y |
| Integration of other WHP programs | 2 | 2 | 0 |  |  |  |  |  |  |  | x |  |  | x |  |  |  |  |  |  |
| Across a variety of settings | 1 | 0 | 1 |  |  |  |  |  |  |  |  |  |  |  |  |  |  |  |  |  |
| Target at workplace needs / Context-aware | 2 | 1 | 1 |  |  |  |  |  |  |  | x |  |  |  |  |  |  |  |  | y |
| Employers can see work conditions | 1 | 1 | 0 |  |  |  |  | x |  |  |  |  |  |  |  |  |  |  |  |  |
| Accurate/ Objective measure of program engagement in real-time | 3 | 1 | 2 |  |  |  |  |  |  |  |  |  |  |  |  | y | x |  |  |  |
| Versatile and multifaceted | 1 | 1 | 0 |  |  |  |  |  |  |  |  |  |  |  |  |  | x |  |  |  |
| Wide Distribution and use of smartphones in population | 9 | 3 | 6 | y |  | y | y |  |  | x |  |  |  |  |  |  | x | y |  |  |
| Wide reach | 10 | 4 | 6 |  |  | y |  |  |  |  | x |  |  |  |  |  |  |  | y | y |
| Independent of SES | 1 | 1 | 0 |  |  |  |  |  |  |  |  |  |  |  |  |  |  |  |  |  |
| High reach of young and female workers | 1 | 1 | 0 |  |  |  |  |  |  | x |  |  |  |  |  |  |  |  |  |  |
| Reach in middle- and low-income countries | 1 | 0 | 1 |  |  |  |  |  |  |  |  |  |  |  |  |  |  |  |  |  |
| Possible for workers who cannot participate in face-to-face activities e.g., shift workers | 3 | 2 | 1 |  |  |  |  |  |  |  |  |  |  |  |  |  |  |  |  |  |
| Preventive use | 2 | 0 | 2 |  |  |  |  |  |  |  |  |  |  |  |  |  | y |  |  | y |
| Little teacher’s guidance needed | 1 | 1 | 0 |  |  |  |  |  |  |  |  |  |  |  |  |  |  |  |  |  |
| Cost-effective | 11 | 7 | 4 |  |  | x |  |  |  |  | x | x |  |  |  | x |  |  |  | y |
| Low costs | 8 | 4 | 4 |  |  |  |  |  |  |  |  |  |  |  |  |  |  |  |  |  |
| Functional and economic benefits | 1 | 1 | 0 |  |  |  |  |  |  |  |  |  |  |  |  |  |  |  |  |  |
| Limits infection risk | 2 | 2 | 0 |  |  |  |  |  |  |  |  |  |  |  |  |  |  |  |  |  |
| Viable | 1 | 1 | 0 |  |  |  |  |  |  |  |  |  |  |  |  |  |  |  |  |  |
| Reduces health disparities | 1 | 0 | 1 |  |  |  |  |  |  |  |  |  |  |  |  |  |  |  |  |  |
| Also, other colleagues may benefit due to cultural change | 1 | 1 | 0 |  |  |  |  |  |  |  |  |  |  |  |  |  |  |  |  | x |
| **Weaknesses** |  |  |  |  |  |  |  |  |  |  |  |  |  |  |  |  |  |  |  |  |
| **Acceptance / Adherence / Use** |  |  |  |  |  |  |  |  |  |  |  |  |  |  |  |  |  |  |  |  |
| Short usage / High attrition | 7 | 6 | 1 |  |  |  |  |  |  |  |  | x |  |  |  |  | y |  | x |  |
| Engagement drops over time | 4 | 2 | 2 |  |  |  |  |  |  |  |  |  |  |  |  |  |  |  |  | y |
| High effort | 1 | 1 | 0 |  |  |  |  |  |  |  |  |  |  |  |  |  |  | x |  |  |
| Lack of time for usage (at work) | 12 | 12 | 0 | x |  |  |  |  | x |  | x |  |  | x |  |  | x |  | x |  |
| Holidays | 1 | 1 | 0 |  |  |  |  |  |  |  |  |  |  |  |  |  |  |  |  |  |
| Additional stressor at work | 3 | 3 | 0 |  |  |  |  |  |  |  |  |  |  |  |  |  |  |  | x |  |
| Difficulty to find space to relax | 1 | 1 | 0 |  |  |  |  |  |  |  |  |  |  |  |  |  | x |  |  |  |
| Integration into daily life | 1 | 1 | 0 |  |  |  |  |  | x |  |  |  |  |  |  |  |  |  |  |  |
| Expectations not fulfilled | 1 | 1 | 0 |  |  |  |  |  |  |  |  |  |  |  |  |  |  |  | x |  |
| One size--fits- all approach | 1 | 1 | 0 |  |  |  |  |  |  |  |  |  |  |  |  |  | x |  |  |  |
| Preferences of private apps | 2 | 2 | 0 |  |  |  |  |  |  |  | x |  |  |  |  |  |  |  |  |  |
| Disliked appearance | 1 | 1 | 0 |  |  |  |  |  |  |  |  |  |  |  |  |  |  |  |  |  |
| Usage dependent on culture and smartphone literacy | 1 | 1 | 0 |  |  |  |  |  |  |  |  |  |  |  |  |  |  |  |  |  |
| Employees need to make choices in the app | 1 | 1 | 0 |  |  |  |  |  |  |  |  |  |  |  |  |  |  |  |  |  |
| Social pressure | 2 | 2 | 0 |  |  |  |  |  |  |  |  |  |  |  |  |  |  |  |  |  |
| Forgot to take phone for a walk | 1 | 1 | 0 |  |  |  |  |  |  |  |  |  |  |  |  |  |  |  |  |  |
| Not always practical, feasible or appropriate to carry the phone | 1 | 1 | 0 |  |  |  |  |  |  |  |  |  |  |  |  |  |  |  |  |  |
| Fear of consequences at work | 1 | 1 | 0 |  |  |  |  |  |  |  |  |  |  |  |  |  |  |  |  |  |
| Privacy and data concern | 5 | 4 | 1 |  |  |  |  | x |  |  | x |  |  |  |  |  |  |  |  | y |
| Lack of perceived benefits | 1 | 1 | 0 |  |  |  |  |  | x |  |  |  |  |  |  |  |  |  |  |  |
| Lack of perceived need | 2 | 2 | 0 |  |  |  |  |  | x |  |  |  |  |  |  |  |  |  |  |  |
| Lack of motivation / Laziness | 2 | 2 | 0 |  |  |  |  |  |  |  |  |  |  |  |  |  |  |  |  |  |
| Younger age did not continue use | 1 | 1 | 0 |  |  |  |  |  |  |  |  |  |  |  |  |  |  |  |  |  |
| Gender Differences | 2 | 2 | 0 |  |  |  |  |  |  |  |  |  |  |  |  |  |  |  |  |  |
| Older population lacks experience with smartphones | 1 | 0 | 1 |  |  |  | y |  |  |  |  |  |  |  |  |  |  |  |  |  |
| **Functionalities** |  |  |  |  |  |  |  |  |  |  |  |  |  |  |  |  |  |  |  |  |
| Reminders perceived as annoying or frustrating | 4 | 4 | 0 |  |  |  |  |  |  |  | x |  |  |  |  |  |  |  | x |  |
| Rewards perceived as unfair | 1 | 1 | 0 |  |  |  |  |  |  |  |  |  |  |  |  |  |  |  |  |  |
| Battery consumption | 1 | 1 | 0 |  |  |  |  |  |  |  | x |  |  |  |  |  |  |  |  |  |
| Technical difficulties (e.g., wireless connection) | 6 | 6 | 0 |  |  |  |  |  |  |  | x |  |  |  | x |  |  |  | x |  |
| Low download rate | 1 | 1 | 0 |  |  |  |  |  |  |  |  |  |  |  |  |  |  |  |  |  |
| Implementation difficulties | 1 | 1 | 0 |  |  |  |  |  |  |  |  |  |  |  |  |  |  |  |  |  |
| No social interaction (e.g., for discussions) | 2 | 2 | 0 |  |  |  |  |  |  |  |  |  |  |  |  |  |  |  |  |  |
| Manual entry was perceived as clunky and inconvenient | 1 | 1 | 0 |  |  |  |  |  |  |  |  |  |  |  |  |  |  |  |  |  |
| System quality | 1 | 1 | 0 |  |  |  |  |  |  |  | x |  |  |  |  |  |  |  |  |  |
| Not installed on private phone | 1 | 1 | 0 | x |  |  |  |  |  |  |  |  |  |  |  |  |  |  |  |  |
| No suitable phone (all platforms need to be included) | 2 | 2 | 0 |  |  |  |  |  | x |  | x |  |  |  |  |  |  |  |  |  |
| **Effectiveness** |  |  |  |  |  |  |  |  |  |  |  |  |  |  |  |  |  |  |  |  |
| Need for more help | 1 | 1 | 0 |  |  |  |  |  |  |  |  |  |  |  |  |  |  |  | x |  |
| Might not be right for severe cases or mental problems (Lack of personal contact) | 3 | 2 | 1 |  |  |  |  |  |  |  |  |  |  |  |  |  |  | y |  |  |
| Issue of measurement accuracy | 3 | 3 | 0 |  |  |  |  |  |  |  | x |  |  |  |  |  |  |  |  |  |
| Self-reported data is not reliable | 1 | 1 | 0 |  |  |  |  |  |  |  |  |  |  |  |  |  |  |  |  |  |
| Onsite perceived more beneficial and useful | 1 | 1 | 0 |  |  |  |  |  |  |  |  |  |  |  |  |  |  |  |  |  |
| Lack of feedback and guidance by a teacher | 1 | 1 | 0 |  |  |  |  |  |  |  |  |  |  |  |  |  |  |  |  |  |
| **Employer perspective** |  |  |  |  |  |  |  |  |  |  |  |  |  |  |  |  |  |  |  |  |
| Heterogenous target group makes development difficult | 1 | 1 | 0 |  |  |  |  |  |  |  |  |  |  |  |  |  |  |  | x |  |
| Lack of intensity and repeatability for long-term effectiveness | 1 | 1 | 0 |  |  |  |  |  |  |  |  |  |  |  |  |  |  |  |  |  |
| Difficult to implement into daily life of healthcare workers | 1 | 1 | 0 |  |  |  |  |  |  |  |  |  |  |  |  |  |  |  |  |  |
| Input from the employer is needed to encourage participation | 1 | 1 | 0 |  |  |  |  |  |  |  |  |  |  |  |  |  |  |  |  |  |
| **Opportunities** |  |  |  |  |  |  |  |  |  |  |  |  |  |  |  |  |  |  |  |  |
| Employer need to support usage | 1 | 1 | 0 |  |  |  |  |  |  |  |  |  |  |  |  |  |  |  |  |  |
| Need to be embedded in other employee support package parts | 2 | 2 | 0 |  |  |  |  |  |  |  |  |  |  |  |  |  |  |  |  |  |
| Connection to other technical devices possible e.g., wearables | 2 | 2 | 0 |  |  |  |  |  |  |  |  |  |  |  |  |  |  |  | x |  |
| Further developments possible (e.g., of reminders and customization) | 1 | 1 | 0 |  |  |  |  |  |  |  |  |  |  |  |  |  |  |  | x |  |
| Future technology developments | 1 | 1 | 0 |  |  |  |  |  |  |  |  |  |  |  |  |  |  |  |  |  |
| Cost-effectiveness | 1 | 1 | 0 |  |  |  |  |  |  |  |  |  |  |  |  |  | x |  |  |  |
| Personalization | 1 | 1 | 0 |  |  |  |  |  |  |  |  |  |  |  |  |  | x |  |  |  |
| Growing number of apps and research | 1 | 1 | 0 |  |  |  |  |  |  |  |  |  |  |  |  |  |  |  |  | x |
| Cultural differences | 1 | 1 | 0 |  |  |  |  |  |  |  |  |  |  |  |  |  |  |  |  |  |
| Refinement based on feedback possible | 1 | 1 | 0 |  |  |  |  |  |  |  |  |  |  |  |  |  |  |  |  |  |
| Interactive support | 1 | 1 | 0 |  |  |  |  |  |  |  |  |  |  |  |  |  | x |  |  |  |
| Refresher sessions needed | 1 | 1 | 0 |  |  |  |  |  |  |  |  |  |  |  |  |  |  |  |  |  |
| Additional functionalities as gamification, challenges, or messages | 2 | 2 | 0 |  |  |  |  |  |  |  |  |  |  |  |  |  |  |  |  |  |
| **Threats** |  |  |  |  |  |  |  |  |  |  |  |  |  |  |  |  |  |  |  |  |
| Without systematic research potential harmful or not effective | 1 | 1 | 0 |  |  |  |  |  |  |  |  |  |  |  |  |  | x |  |  |  |
| Lack of experimental / scientific evidence | 8 | 5 | 3 |  |  |  |  |  |  |  |  | x |  | x |  | x | y |  |  | y |
| Effectiveness dependent on user engagement | 1 | 1 | 0 |  |  |  |  |  |  |  |  |  |  |  |  |  |  |  |  |  |
| Organizational measures are also necessary to complement an app | 1 | 1 | 0 |  |  |  |  |  |  |  |  |  |  |  |  |  |  |  |  |  |
| Limited sustainability of an e-health intervention | 2 | 2 | 0 |  |  |  |  |  |  |  |  |  |  |  |  |  |  |  |  | x |
| Weather conditions influences outdoor activities | 1 | 1 | 0 |  |  |  |  |  |  |  |  |  |  |  |  |  |  |  |  |  |
| Data security needed | 1 | 1 | 0 |  |  |  |  |  |  |  |  |  |  |  |  |  |  |  |  |  |
| Not suitable for clinical conditions | 1 | 1 | 0 |  |  |  |  |  |  | x |  |  |  |  |  |  |  |  |  |  |

^a^X= Factor found in the study

^b^Y= Factor mentioned in the study, referenced to another study
