# Supplementary material for: Advantages and disadvantages of mobile applications for workplace health promotion: A scoping review
Source: PLoS One. 2024 Jan 2;19(1):e0296212. doi: 10.1371/journal.pone.0296212 (PMC10760718; doi:10.1371/journal.pone.0296212)
Supplement: S3 Appendix — (DOCX) [file pone.0296212.s003.docx]

S3 Appendix: Continued Found Strengths, weaknesses, opportunities and threats (2/2)

|  | Billmann et al. (2020) | Bort-Roig et al. (2020) | Emerson et al. (2020) | Haque et al. (2020) | Mascaro et al. (2020) | Morris et al. (2020) | Chan et al. (2021) | Lu et al. (2021) | Morris et al. (2021) | Rich et al. (2021) | Sasaki et al. (2021) | Bartlett et al. (2022) | Bonn et al. (2022) | Deady et al. (2022) | Gnanapragasam et al. (2022) | Hirshberg et al. (2022) | Huberty et al. (2022) | Nuijten et al. (2022) | Puzia et al. (2022) | Rick et al. (2022) | Xu et al. (2022) |
| --- | --- | --- | --- | --- | --- | --- | --- | --- | --- | --- | --- | --- | --- | --- | --- | --- | --- | --- | --- | --- | --- |
| **Strengths** |  |  |  |  |  |  |  |  |  |  |  |  |  |  |  |  |  |  |  |  |  |
| **Acceptance / Adherence / Use** |  |  |  |  |  |  |  |  |  |  |  |  |  |  |  |  |  |  |  |  |  |
| Independent of time and place | y^b^ |  |  |  |  |  |  |  |  | x^a^ |  |  | x |  | x |  |  |  | y | x | x |
| Portable |  |  |  |  |  | y |  |  |  |  |  |  |  |  |  |  |  |  |  |  |  |
| Embedded in daily routines and work life | y |  | y |  |  |  | y |  |  |  |  |  | y |  |  |  |  | x |  |  |  |
| Accessible |  |  |  |  |  | y |  |  | x |  | x | y |  |  | x | x |  |  | y | x |  |
| Easy to access |  |  |  |  |  |  |  |  |  |  |  |  |  |  | x |  |  |  |  |  | x |
| Offline access |  |  |  |  |  |  |  |  |  |  |  |  |  |  |  |  |  |  |  |  |  |
| Short and easy exercises |  |  |  |  |  |  |  |  |  | x |  |  |  |  |  |  |  |  |  |  |  |
| Short usage possible /Brevity |  |  |  |  |  |  |  |  |  |  |  |  |  |  |  |  |  |  |  |  |  |
| Supports taking time to exercise |  |  |  |  |  |  |  |  |  |  |  |  |  |  |  |  |  |  |  |  |  |
| Less time consuming compared to ordinary programs |  |  |  |  | x |  |  |  |  |  |  | x |  |  |  |  |  |  |  |  | x |
| Users are curious (leads to usage) |  |  |  |  |  |  |  |  | x |  |  |  |  |  |  |  |  |  |  |  |  |
| Self-guided (learning) |  |  |  |  |  |  |  |  |  |  | x | y |  |  |  |  |  |  |  |  | x |
| User's Autonomy |  |  |  |  |  |  |  |  |  |  |  |  |  | x |  |  |  | y |  |  |  |
| Control over progress pace |  |  |  |  |  |  |  |  |  |  |  |  |  |  |  |  |  |  |  |  |  |
| Popular technology |  |  |  |  |  |  |  |  |  |  |  | y |  |  |  |  |  |  |  |  |  |
| Interaction with participants in daily life |  |  | y |  |  |  |  |  |  |  |  |  |  |  |  |  |  |  |  |  |  |
| Common topic at work |  |  | x |  |  |  |  |  |  |  |  |  |  |  |  |  |  |  |  |  |  |
| Interpersonal/ peer support |  |  |  |  |  |  |  |  | x |  |  |  |  |  |  |  |  |  |  |  |  |
| Interactive |  |  |  |  |  |  |  |  |  |  |  |  |  |  |  |  |  |  |  |  |  |
| Reduces stigma |  |  |  |  |  |  | y |  |  |  |  |  |  |  |  |  |  |  |  |  |  |
| Anonymity |  |  |  |  |  |  |  |  |  |  |  |  |  |  | x |  |  |  |  |  |  |
| Privacy protection possible |  |  |  |  |  |  |  |  |  |  |  |  |  |  |  |  |  |  |  |  |  |
| Simple, enjoyable, intuitive and interactive design |  |  |  |  |  |  |  |  |  |  |  |  |  |  |  |  |  |  |  |  |  |
| Fun |  |  |  |  |  |  |  |  |  |  |  |  |  |  |  |  |  |  |  |  |  |
| Enjoyment in tackling everyday issues using an app |  |  |  |  |  |  |  |  |  |  |  |  |  |  |  |  |  |  |  |  |  |
| Convenient |  |  |  |  |  |  |  |  |  |  |  |  | x |  |  |  |  |  |  |  | x |
| Simplicity |  |  | y |  |  |  |  |  |  |  |  |  |  |  |  |  |  |  |  | x |  |
| Ease of use |  |  | x |  |  |  |  |  |  |  |  |  |  |  |  |  |  |  |  |  |  |
| User Friendly |  |  |  |  |  |  | y |  |  |  |  |  |  |  |  |  |  |  |  |  |  |
| Scaled at pace |  |  |  |  |  |  |  |  |  |  |  |  |  |  | x |  |  |  |  |  |  |
| Usability of various problems |  |  |  |  |  |  |  |  |  |  |  |  |  |  |  |  |  |  |  |  |  |
| Widespread appeal |  |  |  |  |  |  |  |  |  |  |  |  |  |  |  |  |  |  |  | x |  |
| Cultural factors |  |  |  |  |  |  |  |  | x |  |  |  |  |  |  |  |  |  |  |  |  |
| **Functionalities** |  |  |  |  |  |  |  |  |  |  |  |  |  |  |  |  |  |  |  |  |  |
| Persuasive Features |  |  |  |  |  |  |  |  |  |  |  |  |  |  |  |  |  |  |  |  |  |
| Simple, straightforward functions |  |  |  |  |  |  |  |  |  |  |  |  |  |  |  |  |  |  |  |  |  |
| Various functionalities e.g., gamification, reminders, triggers |  |  |  |  |  |  |  |  |  |  |  |  |  |  |  |  |  |  |  |  |  |
| Multi-component intervention |  |  |  |  |  | x |  |  |  |  |  |  |  |  |  |  |  |  |  |  |  |
| Reminders & Notifications |  |  |  | x |  | x |  |  | x | y |  |  |  |  |  |  |  |  |  |  |  |
| Gamification |  |  |  |  |  |  |  |  |  |  |  |  |  |  |  |  |  | x |  |  |  |
| Competition |  |  | x |  |  |  |  |  |  |  |  |  |  |  |  |  |  |  |  |  |  |
| Sharing of personal progress |  |  | x |  |  |  |  |  |  | y |  |  |  |  |  |  |  |  |  |  |  |
| Integration in other devices |  |  |  |  |  |  |  |  |  |  |  |  |  |  |  |  |  |  |  |  |  |
| Accuracy (Negative and positive) |  |  |  |  |  |  |  |  |  |  |  |  |  |  |  |  |  |  |  |  |  |
| Unobtrusive monitoring |  |  |  |  |  |  |  |  |  |  |  |  |  |  |  |  |  |  |  |  |  |
| Quantified self |  |  | x |  |  |  |  |  |  |  |  |  |  |  |  |  |  |  |  |  |  |
| Visualization |  |  |  |  |  |  |  |  |  |  |  |  |  |  |  |  |  |  |  |  |  |
| Learnability |  |  |  |  |  |  |  |  |  |  |  |  |  |  |  |  |  |  |  |  |  |
| 24 h reachability | y |  |  |  |  |  |  |  |  |  |  |  |  |  |  |  |  |  |  |  |  |
| **Effectiveness** |  |  |  |  |  |  |  |  |  |  |  |  |  |  |  |  |  |  |  |  |  |
| Personalization /Customization |  |  |  |  |  |  | y |  |  |  | y |  |  |  |  | x |  | x |  | x | x |
| Individual choice of content |  |  |  |  |  |  |  |  |  |  |  |  |  |  |  |  |  |  |  |  |  |
| Self-monitoring (also in stressful situations) | y | y | x |  |  |  |  |  | y |  |  |  |  |  |  |  |  |  |  |  |  |
| Automatic and reliable self-monitoring (e.g., sensor-based pedometer) |  |  | x | x |  |  |  |  |  |  | x |  |  |  |  |  |  |  |  |  |  |
| Progress tracking in real time |  |  |  |  |  |  | y |  | y |  |  |  |  |  |  |  |  |  |  |  |  |
| Confidence and reassurance by monitoring |  |  |  |  |  |  |  |  |  |  |  |  |  |  |  |  |  |  |  |  |  |
| Insights into physical activity patterns /awareness |  |  |  |  |  |  |  |  |  |  |  |  |  |  |  |  |  |  |  |  |  |
| Real-time feedback / Ecological momentary intervention |  | y | x |  |  |  |  |  |  |  |  |  |  |  |  |  |  |  |  |  |  |
| Visual feedback |  |  |  |  |  |  |  |  |  |  |  |  |  |  |  |  |  |  |  |  |  |
| Personalized feedback |  |  |  |  |  |  |  |  |  |  |  |  |  |  |  |  |  |  |  |  |  |
| Individual, tailored support for goals and motivation |  |  |  |  |  |  |  |  |  |  |  |  |  |  |  |  |  | y |  | x |  |
| Practice routines can be better established |  |  |  |  |  |  |  |  |  |  |  | x |  |  |  |  |  |  |  |  |  |
| Rigorous |  |  |  |  |  |  |  |  |  |  |  |  |  |  |  |  |  |  |  |  |  |
| Treatment fidelity |  |  |  |  |  |  |  |  |  |  |  |  |  |  |  |  |  |  |  |  |  |
| Effective to prevent depression cases |  |  |  |  |  |  |  |  |  |  |  |  |  | x |  |  |  |  |  |  |  |
| Immediate support |  |  | y |  |  |  |  |  |  |  |  |  |  |  |  |  |  |  |  | x |  |
| High completion rate |  |  |  |  |  |  |  |  |  |  | x |  |  |  |  |  |  |  |  |  |  |
| High engagement | y |  |  |  |  |  |  |  |  |  |  |  |  |  |  |  |  |  |  |  |  |
| Overcomes barriers of ordinary programs e.g., forgot to complete diary |  |  |  | x |  |  |  |  |  |  |  |  |  |  |  |  |  |  |  |  |  |
| Time efficient |  |  |  |  |  |  |  |  |  |  |  |  |  |  |  |  |  |  |  |  |  |
| **Employer perspective** |  |  |  |  |  |  |  |  |  |  |  |  |  |  |  |  |  |  |  |  |  |
| Customization to organization |  |  |  |  |  |  |  |  |  |  |  |  |  |  |  |  |  |  |  |  |  |
| Adaptability | y |  |  |  |  |  |  |  |  |  |  |  |  |  |  |  | x |  |  |  |  |
| Flexibility |  |  |  |  |  |  |  |  |  | x |  |  |  |  |  |  |  |  |  |  | x |
| Integration of other WHP programs |  |  |  |  |  |  |  |  |  |  |  |  |  |  |  |  |  |  |  |  |  |
| Across a variety of settings |  |  |  |  |  |  |  |  | y |  |  |  |  |  |  |  |  |  |  |  |  |
| Target at workplace needs / Context-aware |  |  |  |  |  |  |  |  |  |  |  |  |  |  |  |  |  |  |  |  |  |
| Employers can see work conditions |  |  |  |  |  |  |  |  |  |  |  |  |  |  |  |  |  |  |  |  |  |
| Accurate/ Objective measure of program engagement in real-time |  |  |  |  |  |  |  |  |  |  |  | y |  |  |  |  |  |  |  |  |  |
| Versatile and multifaceted |  |  |  |  |  |  |  |  |  |  |  |  |  |  |  |  |  |  |  |  |  |
| Wide Distribution and use of smartphones in population |  |  |  |  |  | y |  |  | y |  |  |  |  |  | x |  |  |  |  |  |  |
| Wide reach | y | y |  |  |  |  |  |  |  |  |  |  | x |  | x |  |  |  |  | x | y |
| Independent of SES |  |  |  |  |  |  |  |  |  |  |  |  | x |  |  |  |  |  |  |  |  |
| High reach of young and female workers |  |  |  |  |  |  |  |  |  |  |  |  |  |  |  |  |  |  |  |  |  |
| Reach in middle- and low-income countries |  |  |  |  |  |  |  |  |  |  | y |  |  |  |  |  |  |  |  |  |  |
| Possible for workers who cannot participate in face-to-face activities e.g., shift workers |  |  |  |  | y |  |  |  |  |  |  |  |  |  | x |  |  |  |  |  | x |
| Preventive use |  |  |  |  |  |  |  |  |  |  |  |  |  |  |  |  |  |  |  |  |  |
| Little teacher’s guidance needed |  |  |  |  |  |  |  |  |  |  | x |  |  |  |  |  |  |  |  |  |  |
| Cost-effective | y |  |  |  |  |  |  |  | x |  |  |  | x |  |  |  | x |  | y |  | y |
| Low costs |  | y | y |  |  | y |  |  |  | y | x |  |  |  |  | x |  |  |  | x | x |
| Functional and economic benefits |  |  |  |  |  |  |  |  |  |  |  |  |  | x |  |  |  |  |  |  |  |
| Limits infection risk |  |  |  |  |  |  |  |  |  |  |  |  |  |  |  | x |  |  |  |  | x |
| Viable |  |  |  |  |  |  |  |  |  |  |  |  |  |  |  |  | x |  |  |  |  |
| Reduces health disparities |  |  |  |  | y |  |  |  |  |  |  |  |  |  |  |  |  |  |  |  |  |
| Also, other colleagues may benefit due to cultural change |  |  |  |  |  |  |  |  |  |  |  |  |  |  |  |  |  |  |  |  |  |
| **Weaknesses** |  |  |  |  |  |  |  |  |  |  |  |  |  |  |  |  |  |  |  |  |  |
| **Acceptance / Adherence / Use** |  |  |  |  |  |  |  |  |  |  |  |  |  |  |  |  |  |  |  |  |  |
| Short usage / High attrition |  |  |  |  |  |  |  |  | x |  |  | x |  | x |  |  |  |  |  |  | x |
| Engagement drops over time |  |  |  |  |  |  |  |  | y |  |  |  |  |  | x |  |  | x |  |  |  |
| High effort |  |  |  |  |  |  |  |  |  |  |  |  |  |  |  |  |  |  |  |  |  |
| Lack of time for usage (at work) | x |  |  | x |  |  |  |  | x |  |  |  |  |  |  |  | x |  | x |  | x |
| Holidays |  |  |  | x |  |  |  |  |  |  |  |  |  |  |  |  |  |  |  |  |  |
| Additional stressor at work |  |  |  |  |  |  |  |  | x |  |  |  |  |  |  |  |  |  |  |  | x |
| Difficulty to find space to relax |  |  |  |  |  |  |  |  |  |  |  |  |  |  |  |  |  |  |  |  |  |
| Integration into daily life |  |  |  |  |  |  |  |  |  |  |  |  |  |  |  |  |  |  |  |  |  |
| Expectations not fulfilled |  |  |  |  |  |  |  |  |  |  |  |  |  |  |  |  |  |  |  |  |  |
| One size--fits- all approach |  |  |  |  |  |  |  |  |  |  |  |  |  |  |  |  |  |  |  |  |  |
| Preferences of private apps |  |  |  | x |  |  |  |  |  |  |  |  |  |  |  |  |  |  |  |  |  |
| Disliked appearance |  |  |  | x |  |  |  |  |  |  |  |  |  |  |  |  |  |  |  |  |  |
| Usage dependent on culture and smartphone literacy |  |  |  |  |  |  |  |  |  |  | x |  |  |  |  |  |  |  |  |  |  |
| Employees need to make choices in the app |  |  |  |  |  |  |  |  |  |  | x |  |  |  |  |  |  |  |  |  |  |
| Social pressure |  |  |  |  |  |  |  |  | x |  | x |  |  |  |  |  |  |  |  |  |  |
| Forgot to take phone for a walk |  |  | x |  |  |  |  |  |  |  |  |  |  |  |  |  |  |  |  |  |  |
| Not always practical, feasible or appropriate to carry the phone |  |  |  |  |  |  |  |  | x |  |  |  |  |  |  |  |  |  |  |  |  |
| Fear of consequences at work |  |  | x |  |  |  |  |  |  |  |  |  |  |  |  |  |  |  |  |  |  |
| Privacy and data concern |  |  | x | x |  |  |  |  |  |  |  |  |  |  |  |  |  |  |  |  |  |
| Lack of perceived benefits |  |  |  |  |  |  |  |  |  |  |  |  |  |  |  |  |  |  |  |  |  |
| Lack of perceived need |  |  |  | x |  |  |  |  |  |  |  |  |  |  |  |  |  |  |  |  |  |
| Lack of motivation / Laziness |  |  |  | x |  |  |  |  |  |  |  |  |  |  |  |  | x |  |  |  |  |
| Younger age did not continue use |  |  |  | x |  |  |  |  |  |  |  |  |  |  |  |  |  |  |  |  |  |
| Gender Differences |  |  | x |  |  |  |  |  |  |  |  |  |  |  |  |  |  |  |  | x |  |
| Older population lacks experience with smartphones |  |  |  |  |  |  |  |  |  |  |  |  |  |  |  |  |  |  |  |  |  |
| **Functionalities** |  |  |  |  |  |  |  |  |  |  |  |  |  |  |  |  |  |  |  |  |  |
| Reminders perceived as annoying or frustrating |  |  |  |  |  |  |  |  | x |  | x |  |  |  |  |  |  |  |  |  |  |
| Rewards perceived as unfair |  |  |  |  |  |  |  |  |  |  |  |  |  |  |  |  |  | x |  |  |  |
| Battery consumption |  |  |  |  |  |  |  |  |  |  |  |  |  |  |  |  |  |  |  |  |  |
| Technical difficulties (e.g., wireless connection) |  | x |  |  |  |  |  |  |  |  |  | x |  |  |  |  | x |  |  |  |  |
| Low download rate |  |  |  |  |  |  |  |  |  |  |  |  |  |  |  |  | x |  |  |  |  |
| Implementation difficulties |  |  |  |  |  |  |  |  |  |  |  |  |  |  |  |  | x |  |  |  |  |
| No social interaction (e.g., for discussions) |  |  |  | x |  |  |  |  |  |  |  | x |  |  |  |  |  |  |  |  |  |
| Manual entry was perceived as clunky and inconvenient |  |  |  |  |  |  |  |  | x |  |  |  |  |  |  |  |  |  |  |  |  |
| System quality |  |  |  |  |  |  |  |  |  |  |  |  |  |  |  |  |  |  |  |  |  |
| Not installed on private phone |  |  |  |  |  |  |  |  |  |  |  |  |  |  |  |  |  |  |  |  |  |
| No suitable phone (all platforms need to be included) |  |  |  |  |  |  |  |  |  |  |  |  |  |  |  |  |  |  |  |  |  |
| **Effectiveness** |  |  |  |  |  |  |  |  |  |  |  |  |  |  |  |  |  |  |  |  |  |
| Need for more help |  |  |  |  |  |  |  |  |  |  |  |  |  |  |  |  |  |  |  |  |  |
| Might not be right for severe cases or mental problems (Lack of personal contact) |  |  | x |  |  |  |  |  |  |  |  |  |  |  |  |  | x |  |  |  |  |
| Issue of measurement accuracy |  |  | x | x |  |  |  |  |  |  |  |  |  |  |  |  |  |  |  |  |  |
| Self-reported data is not reliable |  |  |  |  |  |  |  |  |  |  |  |  |  |  |  |  |  | x |  |  |  |
| Onsite perceived more beneficial and useful |  |  |  |  |  |  |  |  |  |  |  | x |  |  |  |  |  |  |  |  |  |
| Lack of feedback and guidance by a teacher |  |  |  |  |  |  |  |  |  |  |  | x |  |  |  |  |  |  |  |  |  |
| **Employer perspective** |  |  |  |  |  |  |  |  |  |  |  |  |  |  |  |  |  |  |  |  |  |
| Heterogenous target group makes development difficult |  |  |  |  |  |  |  |  |  |  |  |  |  |  |  |  |  |  |  |  |  |
| Lack of intensity and repeatability for long-term effectiveness |  |  |  |  |  |  |  |  |  |  | x |  |  |  |  |  |  |  |  |  |  |
| Difficult to implement into daily life of healthcare workers |  |  |  |  |  |  |  |  |  |  |  |  |  |  |  |  |  |  |  |  | x |
| Input from the employer is needed to encourage participation |  |  |  |  |  |  |  |  |  | x |  |  |  |  |  |  |  |  |  |  |  |
| **Opportunities** |  |  |  |  |  |  |  |  |  |  |  |  |  |  |  |  |  |  |  |  |  |
| Employer need to support usage |  |  |  |  |  |  |  |  |  |  |  |  |  |  |  |  |  |  | x |  |  |
| Need to be embedded in other employee support package parts |  |  |  |  |  |  |  |  |  |  |  |  |  |  | x |  |  |  |  |  | x |
| Connection to other technical devices possible e.g., wearables |  |  |  |  |  |  |  |  | x |  |  |  |  |  |  |  |  |  |  |  |  |
| Further developments possible (e.g., of reminders and customization) |  |  |  |  |  |  |  |  |  |  |  |  |  |  |  |  |  |  |  |  |  |
| Future technology developments |  |  | x |  |  |  |  |  |  |  |  |  |  |  |  |  |  |  |  |  |  |
| Cost-effectiveness |  |  |  |  |  |  |  |  |  |  |  |  |  |  |  |  |  |  |  |  |  |
| Personalization |  |  |  |  |  |  |  |  |  |  |  |  |  |  |  |  |  |  |  |  |  |
| Growing number of apps and research |  |  |  |  |  |  |  |  |  |  |  |  |  |  |  |  |  |  |  |  |  |
| Cultural differences |  |  |  | x |  |  |  |  |  |  |  |  |  |  |  |  |  |  |  |  |  |
| Refinement based on feedback possible |  |  |  |  |  |  |  |  | x |  |  |  |  |  |  |  |  |  |  |  |  |
| Interactive support |  |  |  |  |  |  |  |  |  |  |  |  |  |  |  |  |  |  |  |  |  |
| Refresher sessions needed |  |  |  |  |  |  |  |  |  |  | x |  |  |  |  |  |  |  |  |  |  |
| Additional functionalities as gamification, challenges or messages |  |  |  | x |  |  |  |  | x |  |  |  |  |  |  |  |  |  |  |  |  |
| **Threats** |  |  |  |  |  |  |  |  |  |  |  |  |  |  |  |  |  |  |  |  |  |
| Without systematic research potential harmful or not effective |  |  |  |  |  |  |  |  |  |  |  |  |  |  |  |  |  |  |  |  |  |
| Lack of experimental / scientific evidence | y |  | x |  |  |  |  |  |  |  |  |  |  |  | x |  |  |  |  |  |  |
| Effectiveness dependent on user engagement |  |  |  |  |  |  |  |  |  |  |  |  |  |  |  |  |  |  | x |  |  |
| Organizational measures are also necessary to complement an app |  |  |  |  |  |  |  |  |  | x |  |  |  |  |  |  |  |  |  |  |  |
| Limited sustainability of an e-health intervention |  |  |  |  |  |  |  |  | x |  |  |  |  |  |  |  |  |  |  |  |  |
| Weather conditions influences outdoor activities |  |  |  | x |  |  |  |  |  |  |  |  |  |  |  |  |  |  |  |  |  |
| Data security needed |  |  |  | x |  |  |  |  |  |  |  |  |  |  |  |  |  |  |  |  |  |
| Not suitable for clinical conditions |  |  |  |  |  |  |  |  |  |  |  |  |  |  |  |  |  |  |  |  |  |

^a^X= Factor found in the study

^b^Y= Factor mentioned in the study, referenced to another study
